# Curcumin and Its Derivatives as Anticancer Agents in Head and Neck Cancer: Molecular Mechanisms and Preclinical Evidence

**DOI:** 10.3390/ijms27125626

**Published:** 2026-06-22

**Authors:** Luana Pinto, João P. N. Silva, Luís Monteiro, Patrícia M. A. Silva

**Affiliations:** 1 UNIPRO-Oral Pathology and Rehabilitation Research Unit, IUCS-CESPU, University Institute of Health Sciences, 4585-116 Gandra, Portugal; a31981@alunos.cespu.pt (L.P.); joaosilva_06@hotmail.com (J.P.N.S.); 2 Associate Laboratory i4HB-Institute for Health and Bioeconomy, University Institute of Health Sciences-CESPU, 4585-116 Gandra, Portugal; 3 UCIBIO-Applied Molecular Biosciences Unit, Translational Toxicology Research Laboratory, 1H-TOXRUN, IUCS-CESPU, University Institute of Health Sciences, 4585-116 Gandra, Portugal

**Keywords:** head and neck cancer, natural compounds, curcumin, curcumin derivatives, anticancer potential

## Abstract

Head and neck cancer (HNC), particularly oral squamous cell carcinoma (OSCC), remains a major clinical challenge due to its aggressive behavior, high recurrence rates, and frequent resistance to conventional therapies. Natural compounds, especially curcumin and its derivatives, have gained increasing attention as potential anticancer agents due to their ability to target multiple molecular pathways involved in tumor progression. This review critically evaluates the current preclinical and translational evidence supporting curcumin and its derivatives as monotherapeutic agents in HNC, with particular emphasis on oral cancer. We integrate the available evidence to assess the biological rationale, therapeutic potential, and current limitations of curcumin-based approaches. The molecular mechanisms underlying their antitumor activity are discussed, including modulation of EGFR/ERK and PI3K/Akt signaling pathways, inhibition of NF-κB and STAT3 activation, induction of apoptosis, regulation of oxidative stress, and suppression of epithelial–mesenchymal transition and tumor invasiveness. In addition, we address the impact of curcumin on the tumor microenvironment and its role in overcoming intrinsic cellular resistance mechanisms. The review also highlights advances in drug delivery strategies, such as nanoformulations, that are designed to improve curcumin’s bioavailability and therapeutic efficacy. By critically integrating current evidence, this review highlights both the promise and the challenges associated with curcumin-based monotherapy in HNC, emphasizing the need for more robust and clinically relevant studies to support future translation.

## 1. Introduction

Head and neck cancer (HNC) is one of the most common malignancies worldwide, placing sixth in global incidence [1]. The majority of these tumors arise from the squamous epithelium and are broadly classified as head and neck squamous cell carcinoma (HNSCC) [2].

Several risk factors contribute to HNC development. Tobacco use, whether through smoking or smokeless forms, remains the most significant and well-established risk factor, often acting synergistically with alcohol consumption to markedly increase carcinogenic potential. Areca nut (betel quid) chewing, highly prevalent in certain regions, is also strongly associated with mucosal damage and malignant transformation. Additional contributors include poor nutritional status, occupational or environmental exposure to dust and particulate matter, and viral infection, particularly with human papillomavirus (HPV), which plays a well-established etiological role in a subset of HNCs, especially oropharyngeal squamous cell carcinomas [3]. This effect is primarily mediated by the viral oncoproteins E6 and E7, which promote degradation of p53 and inactivation of retinoblastoma (Rb), leading to dysregulated cell cycle progression and malignant transformation [4]. Importantly, HPV-positive HNSCC represents a distinct biological and clinical entity, often associated with improved prognosis compared to HPV-negative tumors [5].

Treatment of HNC relies on a multimodal approach that typically includes surgery, radiotherapy, chemotherapy, and, in selected cases, targeted therapies and immunotherapy [5,6,7]. Surgery is generally the first-line treatment for localized disease, aiming to achieve complete tumor resection with histologically negative margins. However, surgery alone is often insufficient for advanced-stage or metastatic disease. In patients presenting with high-risk pathological features, such as positive surgical margins, extracapsular nodal extension, or lymph node involvement, adjuvant radiotherapy, either alone or in combination with chemotherapy (chemoradiotherapy), is commonly recommended to reduce the risk of recurrence [8]. Chemotherapy plays a central role in both curative and palliative settings, with frequently used agents including cisplatin, carboplatin, 5-fluorouracil, docetaxel, and paclitaxel. Targeted therapies, such as cetuximab, an epidermal growth factor receptor (EGFR) inhibitor, may be incorporated into treatment regimens for selected patients. More recently, immune checkpoint inhibitors targeting the programmed cell death 1 (PD-1)/programmed cell death ligand (PD-L) 1 axis, such as pembrolizumab and nivolumab, have emerged as important therapeutic options, particularly in recurrent or metastatic disease, improving overall survival in a subset of patients [9,10].

Resistance to therapy arises through multiple mechanisms that enable tumor cells to evade cytotoxic effects. These include increased drug efflux mediated by membrane transporters, enzymatic inactivation of chemotherapeutic agents, enhanced DNA damage repair, and alterations in drug targets that reduce treatment efficacy [11]. In addition, activation of pro-survival signaling pathways, such as the phosphatidylinositol 3-kinase (PI3K)/Akt signaling pathway, nuclear factor κB (NF-κB) signaling pathway, and mitogen-activated protein kinase (MAPK) signaling pathway, further promotes resistance [12]. Dysregulation of apoptotic pathways also plays a critical role, often through overexpression of anti-apoptotic proteins such as B-cell lymphoma 2 (BCL-2), B-cell lymphoma-extra large (BCL-xL), and survivin [13]. Moreover, epithelial–mesenchymal transition (EMT) contributes to increased invasiveness, metastatic potential, and acquisition of stem-like properties [14]. Resistance is further reinforced by alterations in the tumor microenvironment (TME), including hypoxia and immune evasion, which collectively reduce drug sensitivity [15]. Together, these mechanisms contribute to treatment failure, tumor recurrence, and poor clinical outcomes [5]. Consequently, the 5-year overall survival rate for patients with HNC remains approximately 50%, with only modest improvements in recent decades despite advances in diagnosis and treatment, largely due to late-stage presentation at diagnosis [5].

Natural products have emerged as promising therapeutic candidates, with several studies exploring their ability to modulate EMT and TME signaling pathways. Among these, curcumin and its derivatives have demonstrated significant anticancer potential in preclinical models [16,17]. However, their clinical application is limited by poor absorption, rapid metabolism, and low bioavailability. Although several strategies, including nanoparticle-based delivery systems, have been proposed to overcome these pharmacokinetic limitations, the clinical relevance of curcumin-based approaches remains dependent on the robustness of the underlying biological evidence [17].

This review aims to critically evaluate the current preclinical evidence supporting curcumin and its derivatives as potential monotherapeutic anticancer agents in HNC. Rather than providing a descriptive overview of individual studies, this review examines the biological rationale underlying their anticancer activity by integrating evidence from major oncogenic pathways, including EGFR/ERK, PI3K/Akt, NF-κB, STAT3, apoptosis, oxidative stress, EMT, and TME modulation. Furthermore, it discusses the major challenges limiting clinical translation, including poor bioavailability, formulation-related limitations, and the reliability of experimental models. Particular attention is given to the interpretation of findings derived from cell lines with known authentication issues, including misidentified or cross-contaminated models, as these limitations may influence the robustness and translational relevance of the available evidence (KB cells, HEp-2, CNE-1, CNE-2, HNE-2, HONE-1, SUNE-1, SUNE-2, 5-8F, and 6-10B were excluded from the analysis).

## 2. Chemical Structure and Synthetic Analogs of Curcumin

**Figure 1:**
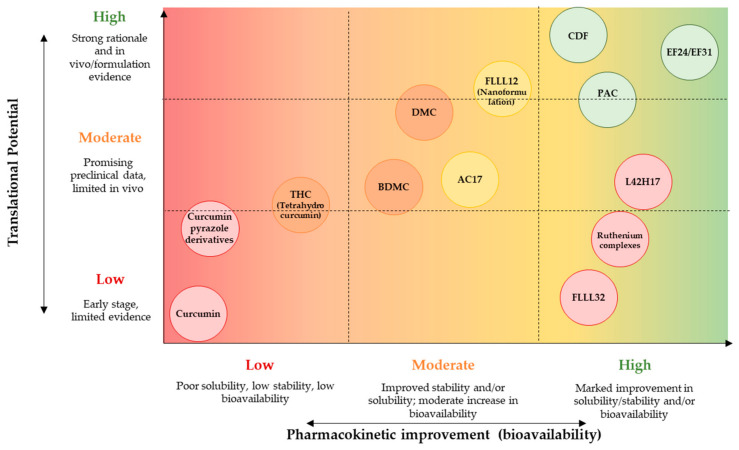
Curcumin derivatives in HNSCC: mechanisms of action, bioavailability and translational potential.

Given the structural diversity and functional heterogeneity of curcumin and its derivatives, these compounds have been organized into distinct groups based on their chemical origin and the primary rationale behind their development, including natural curcuminoids, pharmacokinetically optimized synthetic analogs, and mechanistically designed experimental derivatives. This classification allows a more structured interpretation of the available evidence by distinguishing compounds according to their design strategy and translational relevance, rather than treating them as a homogeneous group of curcumin-related molecules. A summary of the compounds, including their key structural features and reported biological activities, which have been investigated in HNSCC and/or in the management of oral potentially malignant disorders (OPMDs), is provided in Table 1 and Figure 1 to facilitate comparison and improve clarity.

### 2.1. Curcumin and Natural Curcuminoids: Structural Basis and Pharmacological Limitations

Curcumin, chemically identified as 1,7-bis(4-hydroxy-3-methoxyphenyl)-1,6-heptadiene-3,5-dione (1E-6E), is the principal bioactive curcuminoid derived from the rhizome of *Curcuma longa* [18]. It is accompanied in natural extracts by demethoxycurcumin (DMC) and bis-demethoxycurcumin (BDMC), which differ in the degree of methoxy substitution on the aromatic rings (Table 1) [19,20]. Although all three curcuminoids share a common heptadienedione scaffold responsible for their antioxidant and anti-inflammatory properties, curcumin remains the most extensively studied due to its higher abundance and broader biological characterization [21].

From a structural perspective, curcumin contains two substituted aromatic rings linked by a conjugated β-diketone system that underlies its multitarget bioactivity. However, this same structural feature is responsible for its major pharmacokinetic limitations, including chemical instability at physiological pH, rapid metabolic degradation, and poor aqueous solubility. Among natural analogs, DMC and BDMC exhibit slightly improved stability and permeability profiles; however, curcumin generally demonstrates superior antioxidant and anti-inflammatory potency. Overall, the pharmacological limitations of all natural curcuminoids have driven the development of synthetic analogs with improved drug-like properties [21].

Tetrahydrocurcumin, a hydrogenated metabolite of curcumin lacking the α,β-unsaturated carbonyl moiety, is more chemically stable under physiological conditions (0.1 M PBS, pH 7.2, 37 °C) [22]. However, it generally exhibits lower growth-inhibitory activity than curcumin across a wide range of cancer cell lines, including HL60, Ara-C-resistant HL60, A375, A549, HeLa, BGC-823, HCT-116, HEp-2, HT-1080, MCF-7, PC-3, U937, KBM-5, Jurkat, H1299, Calu-6, SCC-4, Panc-1, DU145 and HepG2, and is also less effective in preventing 12-O-tetradecanoylphorbol-13-acetate (TPA)-induced tumor formation, although it shows comparable in vivo efficacy in MDA-MB-231 models [23,24,25,26,27,28]. Moreover, curcumin induced higher ROS production, reduced cell motility in human gingival fibroblasts (HGFs) and human submandibular gland carcinoma (HSG) cells, and promoted nuclear accumulation of nuclear factor erythroid 2-related factor 2 (Nrf2), whereas tetrahydrocurcumin did not [29,30]. Despite its lower cytotoxicity, tetrahydrocurcumin has demonstrated superior effects in certain contexts, including radiosensitization of glioma cells, inhibition of azoxymethane-induced colon carcinogenesis, enhanced antiangiogenic activity in hepatocellular carcinoma models, and improved survival in H22 ascites tumor-bearing mice [31,32,33,34].

### 2.2. Synthetic Analogs Designed to Improve Pharmacokinetic Properties

Synthetic curcumin analogs represent a continuum of rationally designed modifications aimed at overcoming the intrinsic pharmacokinetic limitations of curcumin while, in some cases, enhancing biological activity [35]. These compounds predominantly target the β-diketone moiety or introduce conformational constraints to reduce metabolic susceptibility.

Diphenyldifluoroketone (EF24) and 3,5-Bis(2-pyridinylmethylidene)-4-piperidone (EF31) are representative monoketone analogs that exhibit enhanced chemical stability and improved pharmacokinetic profiles compared with curcumin [36]. Both EF24 and EF31 demonstrate potent inhibition of pro-inflammatory mediators, including NF-κB signaling and cytokine production (TNF-α, IL-1β and IL-6), and display increased cytotoxicity across multiple cancer models [36]. EF24, in particular, has been widely reported to suppress VEGF expression and exhibit broad-spectrum anticancer activity across diverse tumor types, including OSCC [37,38,39,40,41,42,43,44,45,46]. In parallel, EF31 shows an improved pharmacokinetic profile, enhanced cytotoxicity in colorectal and pancreatic cancer cells, and increased antiangiogenic activity compared to curcumin [47,48,49,50].

Similarly, the curcumin-difluorinated (CDF) analog incorporates fluorine substitutions that enhance tissue distribution, metabolic stability, and systemic exposure [51,52]. Preclinical studies indicate improved activity in cancer stem cell (CSC) models [53], inhibiting the growth of multiple human tumor cell lines [54,55,56] and attenuating cisplatin-induced renal toxicity in rat models [55]. In the non-small cell lung cancer (NSCLC) cell lines A549 and H1299, CDF suppressed matrix metalloproteinase (MMP)-2 expression and activity more effectively than curcumin, suggesting a stronger inhibitory effect on invasion and metastatic potential [57]. Despite these improvements, CDF still presents limitations in aqueous solubility and metabolic stability, which continue to hinder clinical translation [58].

Other derivatives, including CLEFMA, FLLL12, FLLL32, PAC, and GO-Y078, follow similar rational design principles, consistently demonstrating improved physicochemical properties and enhanced antiproliferative effects in preclinical cancer models. Collectively, this group illustrates that pharmacokinetic optimization is a primary driver in curcumin analog development, although clinical validation remains largely absent.

CLEFMA is a 3,5-bis(benzylidene)-4-piperidone derivative bearing an N-maleic acid functional group and a 2-chloro substitution on its aromatic rings [59]. Compared with curcumin, CLEFMA exhibits improved solubility and bioavailability [60]. Moreover, it has demonstrated greater antiproliferative activity than curcumin in the lung cancer cell line H441 [61,62]. FLLL12 and FLLL32 are obtained through modifications of the aryl side chain of curcumin [63]. FLLL12 has demonstrated greater efficacy than curcumin across multiple cancer cell lines, including lung, breast, prostate, pancreatic, HNC, and colorectal cancers [63,64,65,66,67]. It also exhibits more favorable pharmacokinetic parameters (C_max_ and AUC) in mice [63]. FLLL32 is a curcumin-derived JAK2/STAT3 inhibitor in which the two hydrogen atoms on the central carbon are replaced with spiro-cycloalkyl rings, stabilizing the diketo form and preventing enolization and thereby enhancing chemical stability [68]. FLLL32 has shown greater potency than curcumin in inducing apoptosis in melanoma cells and increased cytotoxicity across multiple cancer types, including osteosarcoma, breast, colorectal, glioblastoma, rhabdomyosarcoma, multiple myeloma, and liver cancers [69,70,71,72,73]. Additionally, FLLL32 has also demonstrated greater efficacy against colon cancer stem-like cells [74]. GO-Y078, a diarylpentanoid analog, exhibited a lower GI_50_ than curcumin in multiple myeloma cells, along with improved solubility and stronger inhibition of ABCG2 activity [75,76,77]. 4-hydroxy-3-methoxybenzylidene)-N-methyl-4-piperidone (PAC) is a curcumin analog that exhibits greater chemical stability than curcumin in both PBS and circulating blood [78]. PAC is less lipophilic and demonstrates improved water solubility [79]. In addition, PAC was found to induce apoptosis more effectively than curcumin in colon and breast cancer cells and to inhibit tumor growth more efficiently in vivo [79,80]. Also, MTH-3 exhibits improved water solubility, enhanced bioavailability, and stronger inhibitory effects against triple-negative breast cancer compared to curcumin [81,82]. Mono-O-demethylcurcumin is a curcumin analog that was found to have a lower IC_50_ in an OSCC cell line [83].

### 2.3. Mechanistically Optimized and Highly Experimental Analogs

A second category of derivatives has been developed with the aim of enhancing specific molecular mechanisms of action rather than primarily addressing pharmacokinetic constraints. These compounds often exhibit potent but context-dependent biological effects in preclinical systems.

AC17, for example, a 4-arylidene curcumin analog, has been described as an inhibitor of IkB kinase (IKK) signaling and has been linked to NF-κB suppression and p53 reactivation in cancer models [84]. In OSCC, its activity has additionally been associated with modulation of Forkhead box O (FOXO3) signaling [85]. Nonetheless, evidence remains largely restricted to in vitro systems [85,86].

Other structurally diverse analogs, including dibenzylideneacetones (DBAs) [87,88], thiopyranone dioxides [89,90], and trienone derivatives [91,92,93,94], demonstrate enhanced cytotoxic or pathway-specific activity in selected cell line models. However, these compounds are typically characterized by limited pharmacological characterization, restricted model systems, and an absence of in vivo or clinical validation. Notably, several monoketone and diarylpentanoid derivatives, including the difluorodiarylidenyl piperidone (H-4073) [95,96], the diarylidenylpieperidone HO-3867 [95,97,98], the mono-carbonyl L42H17 [99], and L48H37 [100,101,102], have shown increased antiproliferative activity in cancer cell lines. Nevertheless, their translational relevance remains uncertain due to incomplete toxicological profiling and limited pharmacokinetic data.

Curcumin pyrazole is a diketo-modified analog that exhibits enhanced anti-inflammatory activity and greater enzyme selectivity in preclinical models [103,104,105,106,107]. However, it is also associated with lower gastrointestinal absorption and higher predicted toxicity [108].

Curcumin–triterpene hybrids are synthesized through the covalent conjugation of curcumin with pentacyclic triterpenoids, such as oleanolic, ursolic, or betulinic acids [109]. This molecular hybridization strategy aims to enhance antitumor activity and optimize the physicochemical properties of both phytochemicals by exploiting their synergistic effects. However, curcumin showed higher anticancer activity in several cancer cell lines than curcumin–triterpene hybrids [107,109].

Across all classes of curcumin derivatives, a recurring pattern emerges: structural modification frequently enhances in vitro potency and, in some cases, improves pharmacokinetic behavior; however, these advantages have not yet translated into robust clinical evidence. Importantly, most findings are derived from single-cell line models or isolated preclinical systems, limiting generalizability. Furthermore, variability in experimental conditions, lack of standardized comparative frameworks, and absence of clinical validation collectively constrain the translational interpretation of the available data. Overall, while curcumin derivatives represent a chemically diverse and biologically active class of compounds with significant anticancer potential, their current evidence base remains predominantly preclinical. Future studies should prioritize standardized comparative evaluations, in vivo validation, and pharmacokinetic–pharmacodynamic integration to better define their translational relevance in oncology.

**Table 1:** Comparison of curcumin derivatives and analogs: therapeutic potential and drawbacks.

	Compound	Structural Strategy	Main Mechanism of Action	Main Advantages	Main Disadvantages	References
Natural Curcuminoids	Curcumin 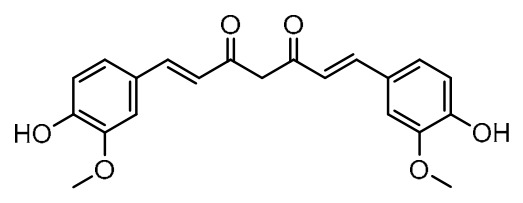	Natural diarylheptanoid polyphenol	NF-κB/STAT3 inhibition; PI3K/Akt and EGFR modulation; apoptosis induction; anti-inflammatory effects	Broad biological activity; multitarget anticancer potential	Poor solubility, low bioavailability, rapid metabolism	[18,21]
	Demethoxycurcumin (DMC) 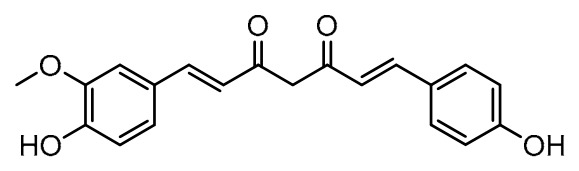	Removal of one methoxy group	NF-κB inhibition; apoptosis induction; oxidative stress modulation	Improved solubility and bioavailability compared with curcumin	Lower antioxidant activity and faster metabolism	[20,21]
	Bisdemethoxycurcumin (BDMC) 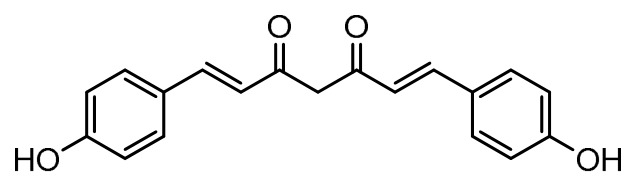	Removal of both methoxy groups	Apoptosis induction; NF-κB modulation; antiproliferative effects	Improved permeability and bioavailability	Lower antioxidant potency and shorter half-life	[20,21]
	Tetrahydrocurcumin (THC) 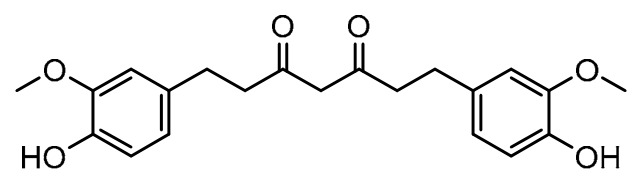	Hydrogenated curcumin analog	Antioxidant activity; anti-inflammatory effects; cell cycle and apoptosis regulation	Higher chemical stability under physiological conditions	Lower anticancer activity in most in vitro models	[23,24,25,26,27,28,29,30,31,32,33,34]
Synthetic Curcuminoids and Derivatives	AC17 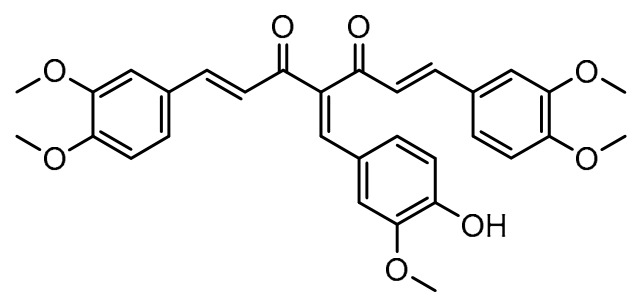	Monocarbonyl curcumin analog	IKK/NF-κB inhibition; apoptosis induction	Improved bioavailability, metabolic stability, and anticancer activity	No clinical validation	[84,85,86]
	CLEFMA 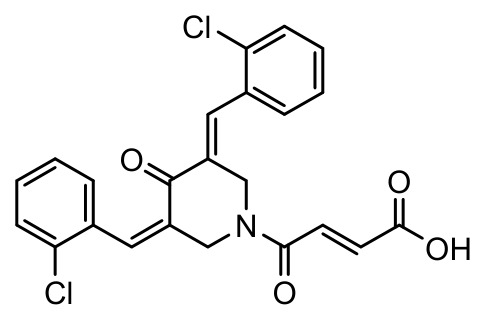	Modified curcumin derivative	NF-κB modulation; inhibition of proliferation and survival pathways	Improved solubility and bioavailability	Limited pharmacokinetic and safety data	[60,61,62]
	Curcumin-difluorinated (CDF) 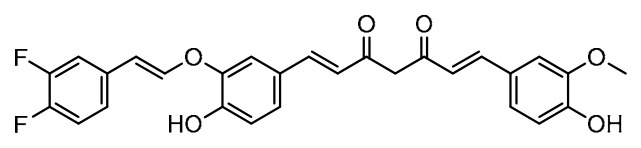	Difluorinated analogue	NF-κB inhibition; cancer stem cell pathway modulation; apoptosis induction	Enhanced tissue distribution and prolonged longer half-life	Poor solubility, metabolic instability	[51,52,53,54,55,56,57,58]
	Curcumin-pyrazole 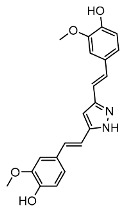	Pyrazole-based heterocyclic modification	COX pathway modulation; anti-inflammatory activity; cytotoxic effects	Increased COX selectivity and anti-inflammatory activity	Lower gastrointestinal absorption, higher predicted toxicity	[103,104,105,106,107,108]
	Curcumin–triterpene hybrid 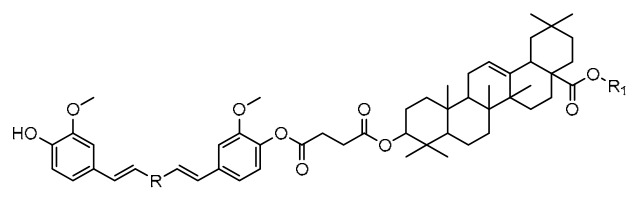	Curcumin–triterpene hybridization	Apoptosis induction; modulation of survival pathways	Theoretical synergistic design strategy	Lower anticancer activity than curcumin in preclinical models	[107,109]
	Dibenzylideneacetone (DBA) 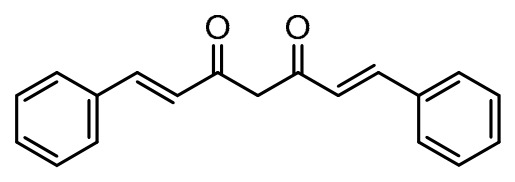	Simplified curcumin analog with Michael acceptor	Apoptosis induction; inhibition of tumor survival signaling	High structural stability, potent cytotoxicity, more effective Michael acceptor	Lower anticancer activity than curcumin in preclinical models; limited in vivo validation	[87,88]
	EF24 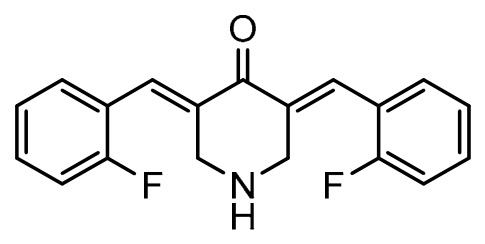	Monocarbonyl curcumin analog	NF-κB inhibition; inflammatory cytokine suppression; ROS-mediated apoptosis	Higher stability and improved pharmacological profile	Limited clinical validation and toxicity profiling	[68]
	EF31 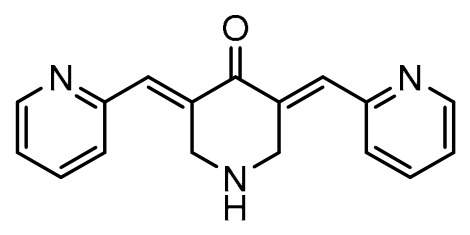	Stabilized monocarbonyl analog	NF-κB pathway inhibition; apoptosis induction; antiangiogenic effects	Enhanced potency and improved pharmacokinetic properties	Limited translational evidence	[46,57,58,59,60]
	FLLL12 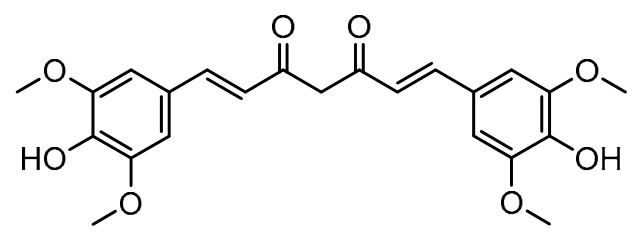	Difluorinated curcumin analog	STAT3 inhibition; suppression of survival signaling; apoptosis induction	Increased cytotoxicity and improved PK profile	Limited clinical validation	[63,64,65,66,67]
	FLLL32 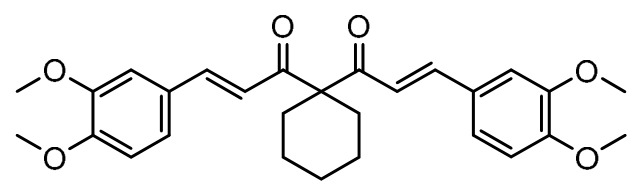	STAT3-targeted curcumin analog	JAK2/STAT3 inhibition; apoptosis induction	Greater stability and potency	Limited in vivo and toxicity data	[68,69,70,71,72,73,74]
	GO-Y078 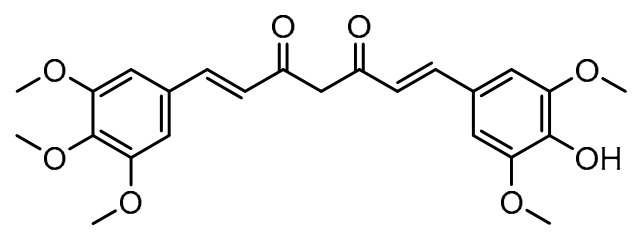	Solubility-enhanced analog	ABCG2 inhibition; apoptosis induction	Improved solubility and reduced GI50 values	Complex synthesis, safety profile not fully established	[75,76,77]
	H-4073 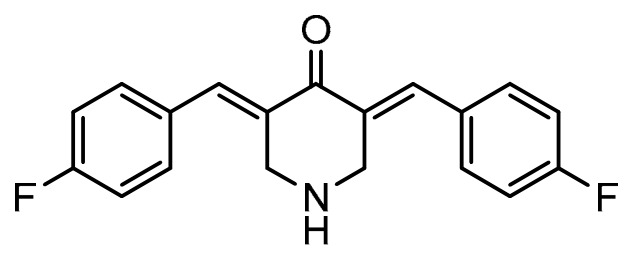	Synthetic curcumin analog	Cytotoxicity induction; apoptosis-related signaling; inhibition of JAK/STAT3, FAK, Akt and VEGF signaling pathways	Enhanced cellular uptake and cytotoxicity	Limited in vivo data	[95,96]
	HO-3867 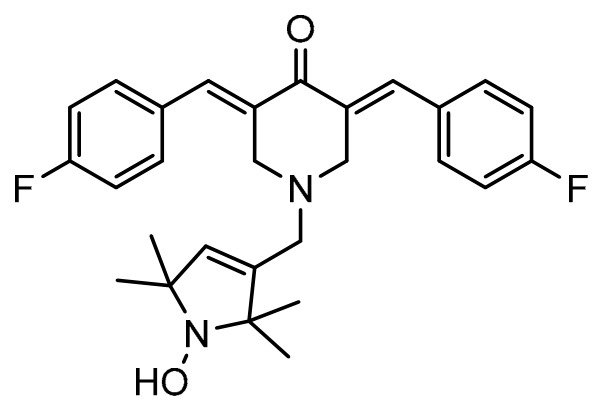	Uptake-optimized analog	ROS-mediated apoptosis; STAT3 pathway modulation	Enhanced cellular uptake and greater cytotoxicity	Limited translational evidence	[95,97,98]
	L42H17 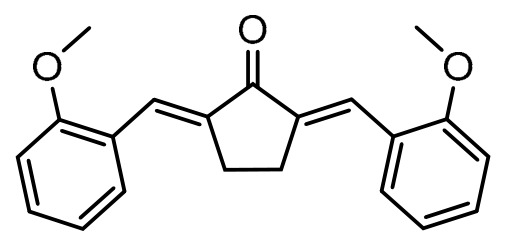	Synthetic stabilized analogs	Cell cycle arrest; apoptosis induction	Improved stability and enhanced anticancer activity	Limited pharmacokinetic and in vivo validation	[99,100,101,102]
	L48H37 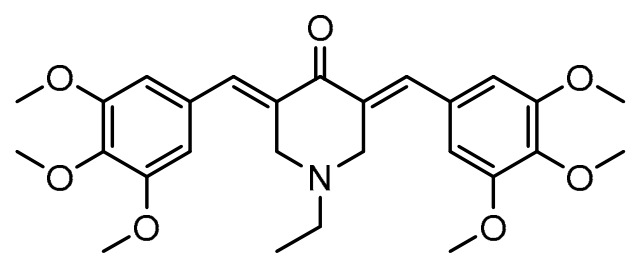					
	Mono-O-demethylcurcumin 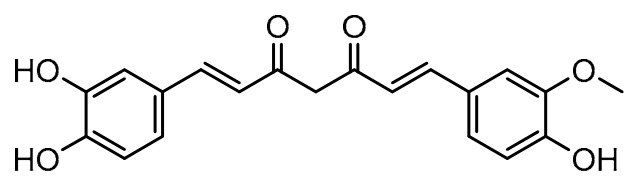	Demethylated derivative	Apoptosis induction; inhibition of proliferation pathways	Lower IC_50_ in an OSCC cell line	Limited mechanistic and in vivo validation	[83]
	MTH-3 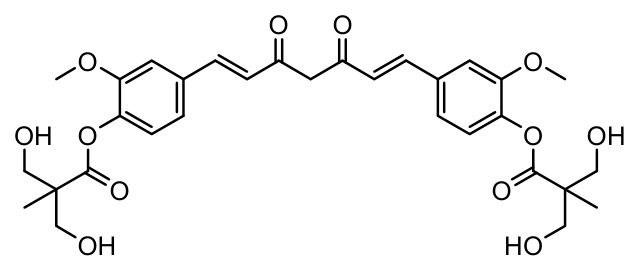	Bioavailability-improved derivative	Apoptosis induction; modulation of tumor progression pathways	Improved solubility, bioavailability and anticancer activity	Limited clinical validation	[81,82]
	PAC 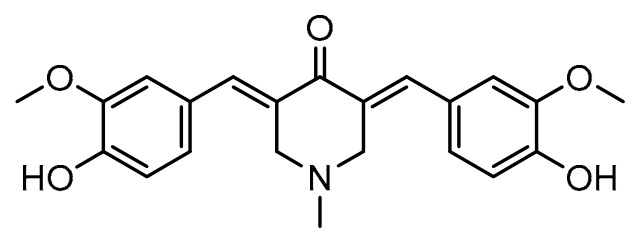	Phosphorylated curcumin derivative	NF-κB inhibition; anti-inflammatory effects; apoptosis induction	Improved stability and solubility; enhanced apoptosis and tumor inhibition in vivo	Limited clinical evidence	[78,79,80]
	Thiopyranone dioxide 41 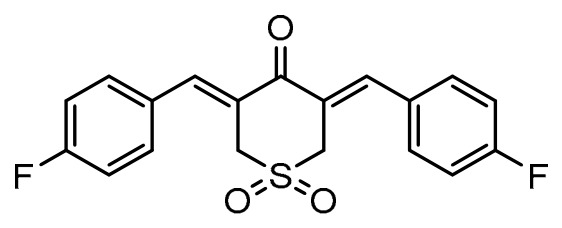	Thiopyranone scaffold modification	Antiproliferative activity; apoptosis induction	Increased hydrolytic stability, antiproliferative activity	Limited in vivo and clinical data	[89,90]
	Thiopyranone dioxide 227 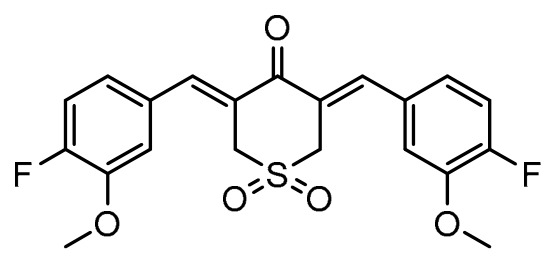					
	Trienone 4 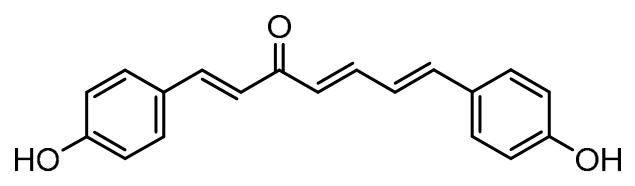	Modified conjugated trienone structure	Mitochondrial apoptosis; ROS modulation	Activity against resistant OSCC models	Variable toxicity in non-cancerous cells	[91,92,93,94]
	Trienone 11 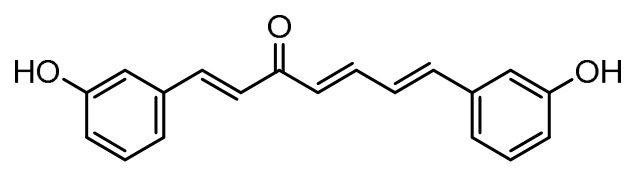					

## 3. Key Molecular Targets of Curcumin and Derivatives

### 3.1. Modulation of EGFR/ERK and PI3K/Akt Signaling

**Figure 2:**
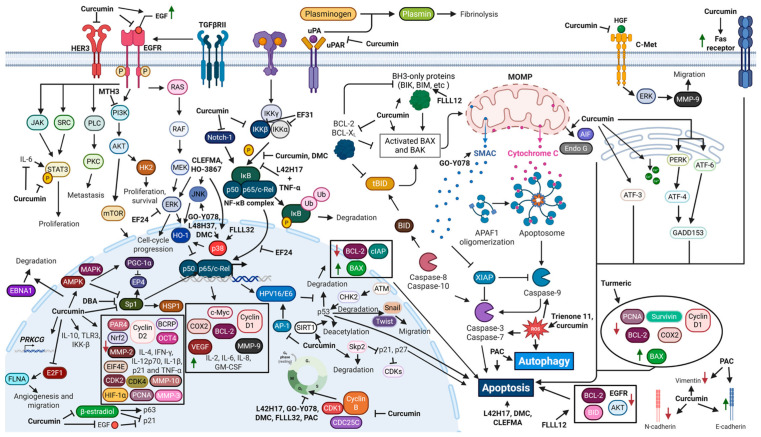
Targeted pathways by curcumin and its analogs in oral cancer. Curcumin exerts its anticancer activity by inhibiting EGFR and HGF signaling, E2F1, Sp1, AP-1 and β-estradiol activity and by repressing NF-κB nuclear translocation and activity. DBA also represses Sp1. Moreover, curcumin decreases the expression of proteins involved in migration, survival and proliferation, such as vimentin, N-cadherin, uPA, MMP-2, CDK2 and BCL-2, while increasing the expression of pro-apoptotic BCL-2 family proteins and E-cadherin, indicating migration repression. Curcumin also promotes autophagy by increasing ROS production and apoptosis by promoting ATF-3, ATF-6, AIF, Endo G, FAS and PERK expression, SIRT-1 activity and Skp2 degradation. Curcumin also leads to cell arrest at the G2/M phase by inhibiting CDK1, CDC25C and cyclin B. MTH3 inhibits PI3K, repressing cell proliferation, while DBA inhibits Sp1, leading to reduced NF-κB activity. GO-Y078, L48H37 and DMC promote JNK and p38 activity, while CLEFMA and HO-3867 also promote ERK. FLLL32 only promotes p38 resulting in NF-κB repression. EF24 represses ERK, but also NF-κB nuclear translocation. Curcumin, DMC, EF31 and L42H17 + TNF-α prevent the phosphorylation of IκB and consequently NF-κB nuclear translocation. In addition, L42H17, GO-Y078, DMC, FLLL32 and PAC promoted cell arrest at the G2/M phase. Trienone 11 promotes ROS production, inducing autophagy. PAC also induces autophagy but also apoptosis. Furthermore, FLLL32, GO-Y078, L42H17, DMC, CLEFMA and turmeric also promote apoptosis, while PAC decreases vimentin expression and increases E-cadherin, repressing invasion. The green arrow represent upregulation while the red arrows represent downregulation.

Curcumin exerts its cancer effects through the modulation of multiple interconnected intracellular signaling pathways involved in proliferation, survival and therapy resistance (Figure 2). Network-based analyses in OSCC have identified several central hub genes associated with curcumin activity, including *VEGFA*, *AKT1*, *TNF*, hypoxia-inducible factor 1α (*HIF-1α*), *EGFR*, *JUN*, signal transducer and activator of transcription 3 (*STAT3*), *MMP-9*, *EGF*, and *MAPK3*, highlighting the broad regulatory network targeted by curcumin in this malignancy [110]. Among these, the EGFR/extracellular signal-regulated kinase (ERK) and PI3K/Akt axes are particularly relevant, as they represent key drivers of tumor growth and resistance to therapy [111].

EGFR is a member of the tyrosine kinase family of growth factor receptors. EGFR triggers multiple downstream signaling cascades, including RAS/RAF/MEK/ERK, PI3K/Akt/mTOR, PLC-γ1/protein kinase C (PKC), SRC, JNK, and JAK/STAT pathways, which collectively regulate cell proliferation, survival, differentiation, and division [112].

In OSCC models, curcumin consistently interferes with these signaling networks, primarily by inhibiting EGFR phosphorylation and suppressing downstream mediators such as Akt, ERK1/2, and STAT3, without altering total EGFR expression. However, context-dependent effects have also been reported, including EGFR activation under specific experimental conditions, suggesting pathway complexity and possible feedback regulation [113].

At the level of PI3K/Akt/mTOR signaling, curcumin induces cell cycle arrest and reduces tumor cell viability through coordinated downregulation of EGFR, PI3K, Akt, mTOR, and PD-L1 in HNSCC cell lines, including SCC-9 [114]. This is accompanied by suppression of key translational regulators such as EIF4E, S6K, and RICTOR, indicating inhibition of both mTORC1 and mTORC2 complexes.

In the FaDu and Cal27 cell lines, curcumin reduces proliferation and migration while inducing apoptosis, effects associated with downregulation of EGFR, STAT3, COX-2, cyclin D1, c-Myc, BCL-2, and BCL-xL [115]. Notably, these effects are further enhanced when combined with the EGFR/STAT3 inhibitor AG490, supporting the involvement of EGFR/STAT3 cross-talk in curcumin’s mechanism of action.

Additional evidence from SAS cells (human tongue squamous cell carcinoma) and OECM1 cells demonstrates that curcumin downregulates EGFR and the efflux transporter BCRP while also reducing Akt activation and oxidative stress-related signaling via Nrf2 modulation, suggesting broader effects on both survival and drug resistance mechanisms [116].

Overall, curcumin modulates EGFR/ERK and PI3K/Akt signaling in HNSCC through a multitargeted mechanism that converges on suppression of proliferation, survival, and migration pathways. While experimental evidence is consistent across multiple models, variability in EGFR activation patterns highlights the context-dependent nature of curcumin signaling effects.

### 3.2. Inhibition of NF-κB, STAT3, and Survival Pathways

NF-κB and STAT3 are key transcription factors that play central roles in regulating cell survival, inflammation, proliferation, invasion, and therapeutic resistance in HNSCC. These pathways are frequently activated in tumor cells and converge on the regulation of genes such as COX-2, cyclin D1, BCL-2 family members, IL-6, and MMP-9, thereby promoting tumor progression and resistance to apoptosis [117,118].

A substantial body of preclinical evidence indicates that curcumin can modulate the canonical NF-κB signaling cascade, mainly through inhibition of IKK activity, preventing IκBα phosphorylation and degradation and thereby reducing NF-κB nuclear translocation and transcriptional activity [119]. This upstream inhibition leads to the downregulation of multiple survival and inflammatory mediators, including cyclin D1, COX-2, IL-6, IL-8, and BCL-2 family proteins, resulting in reduced proliferation and increased apoptosis across HNSCC models.

In OSCC cell lines, curcumin has been shown to suppress proliferation by downregulating specific protein 1 (Sp1) and subsequent inhibition of p65 and heat shock factor 1 (HSF1) expression, leading to reduced NF-κB activity [120]. Curcumin was also shown to reduce the expression of prostaglandin E2 receptor 4 (EP4), a receptor involved in cell growth and invasion, through modulation of AMPKα/p38 MAPK and Sp1 signaling, resulting in reduced viability of Tu212LN and SCC-38 cells [121]. However, some mechanistic studies relied on cell lines with reported authentication concerns, including HEp-2 and CNE-1, which limits interpretation of these findings [121,122,123]. Similarly, although Tu212LN remains classified as an HNSCC model, its reported STR similarity with other cell lines highlights the importance of considering cell line validation when interpreting preclinical data [124].

Additional studies in Cal27 and other HNSCC models confirm inhibition of NF-κB signaling associated with decreased expression of cyclin D1, MMP-9, VEGF, and anti-apoptotic proteins, as well as induction of G2/M cell cycle arrest [125,126,127]. Inhibition of NF-κB was also observed in the PCI-1 and BHY cell lines, leading to decreased levels of IL-6 and IL-8. In the BHY cell line, IL-2, IL-4 and granulocyte–macrophage colony-stimulating factor (GM-CSF) were also downregulated, while IL-10 was upregulated and c-Myc levels reduced in the PCI-1 cell line. Both cell lines showed reduction of Toll-like receptor (TLR) 3, IKK-β, IκBα and cyclin D1 after curcumin treatment [128].

In the salivary cells of HNSCC patients and in the UM-SCC1 cell line, curcumin inhibited IKK-β activity and reduced several inflammatory cytokines, although effects on HNSCC patients were modest and may require higher concentrations to achieve relevant pathway inhibition [129]. Similarly, inhibition of IKK activity in MDA-686LN cells reduced NF-κB activation and downstream targets such as cyclin D1, IL-6, COX-2, and MMP-9 [130,131].

Beyond NF-κB, curcumin also interferes with STAT3 signaling. In HNSCC models, it inhibits STAT3 phosphorylation and nuclear translocation, suppressing IL-6-mediated activation and reducing downstream proliferative signaling. In some contexts, curcumin demonstrated greater inhibitory potency on STAT3 activation than classical pathway inhibitors, highlighting its multitarget activity [132].

Additional evidence suggests that curcumin also interacts with upstream regulators of inflammatory signaling, including TNF-α and Notch-1 pathways. In HPV16-positive oral cancer cells, curcumin suppressed AP-1 and NF-κB activity, reducing E6 oncoprotein expression, stabilizing p53, and promoting apoptosis through modulation of BCL-2 family protein [133,134].

In the p63-positive salivary duct adenocarcinoma cell line A253, curcumin also modulated TNF-α/NF-κB-related signaling, reducing p63 expression, increasing p21 levels, and limiting migration-associated responses [135]. Similarly, the curcumin analog L42H17 inhibited NF-κB pathway activation in FaDu cells by reducing IKK phosphorylation and increasing IκBα levels. L42H17 induced G2/M arrest and apoptosis through modulation of cyclin B1, CDK1, BCL-2, BAX, caspase-3, and PARP cleavage, and reduced tumor volume more effectively than curcumin in vivo [99].

Moreover, two ruthenium-enhanced curcumin derivatives have been evaluated in HNSCC cell lines. Complex 3 displayed higher cytotoxicity and stronger inhibition of cell migration, along with greater repression of IKK-β, reduced IL-8 expression, and more pronounced inhibition of NF-κB nuclear translocation and activity compared with complex 4. In contrast, complex 4 showed a slightly superior ability to inhibit colony formation. Both complexes were more effective than curcumin at inhibiting NF-κB and IKK-β; however, only complex 3 was more effective than curcumin in reducing IL-8 expression and NF-κB nuclear translocation, whereas curcumin remained more effective at reducing IL-6 levels [136].

Using the Tu212 cell line, which has been reported to share an identical STR profile with other HNSCC cell lines, EF24 and EF31 markedly reduced cell viability in vitro. In vivo, EF31 reduced tumor volume and decreased NF-κB p65 expression and phosphorylation, as well as IKK-α and IKK-β phosphorylation, while increasing total IKK-β protein levels and IκBα phosphorylation and reducing IκBα protein levels [124,137].

Furthermore, DMC reduced cell viability and increased apoptosis by reducing NF-κB activation and nuclear translocation, leading to decreased BCL-2 and BCL-xL expression; increased BAX and BCL-2-associated death promoter (BAD) levels; and activated PARP and caspases-3, -8 and -9 in the FaDu cell line [138].

In SCC-4 cells, curcumin, DMC and BDMC led to higher proliferation inhibition than tetrahydrocurcumin and turmerones. However, the authors suggest that repression of cell proliferation and inhibition of NF-κB by curcuminoids are not strictly correlated [28].

In addition, several curcumin analogs were synthesized and evaluated in oral cancer cell lines, leading to decreased cell viability. Analogs 2 and 5 inhibited STAT3, while 8 affected Akt and focal adhesion kinase (FAK) activation. Nonetheless, none of the compounds investigated interfered with ERK1/2 phosphorylation [139].

Overall, available preclinical evidence suggests that curcumin and its derivatives exert anticancer effects in HNSCC through modulation of NF-κB and STAT3 signaling pathways and their downstream survival networks, together with regulation of associated inflammatory, survival, and apoptotic networks. These findings support the biological rationale for curcumin-based strategies; however, the evidence remains largely derived from in vitro models, with limited in vivo validation and no definitive clinical confirmation.

### 3.3. Modulation of Apoptosis and Autophagy

Building on its effects on NF-κB and survival signaling pathways, curcumin also modulates key cellular processes involved in programmed cell death, including apoptosis and autophagy.

For instance, curcumin and the curcumin analogs trienone 4 and trienone 11 were found to induce cell death of both a wild type OSCC cell line and a multidrug resistant OSSC cell line. Trienone 11 showed the lowest IC_50_ value of the compounds tested and induced cell death by promoting ROS production, leading to activation of caspases-3, -7 and -9 [94]. Recently, curcumin was also shown to trigger cell death, at least in part, by increasing ROS production. It also promoted cell death in 3D spheroids, hindering their structural integrity while repressing tumor growth in vivo [140].

Moreover, curcumin decreased cell survival more effectively than isoeugenol, biseugenol, eugenol-dimer, vanillin and eugenol, while showing comparable efficacy to α-diisoeugenol in both HGF and HSG cells [141,142]. It was also the second most effective compound in scavenging 1,1-diphenyl-2-picrylhydrazyl radicals, only surpassed by eugenol-dimer, and demonstrated strong inhibition of free-radical-mediated polymerization, suggesting that curcumin is a strong radical scavenger [142]. At lower concentrations, curcumin induced greater ROS production and increased the proportion of cells in early apoptosis, with these effects being more pronounced in HGF than in HSG cells. At higher concentrations, ROS production was greater in HSG cells, whereas early apoptosis was minimal in both cell lines. In addition, curcumin reduced mitochondrial membrane potential in both cell types, with a more pronounced effect observed in HGF cells [141]. On the other hand, a similar study reported that curcumin induced higher ROS production in the HSG cell line. All tested ROS inhibitors, with the exception of superoxide dismutase (SOD), attenuated curcumin-induced ROS generation, which may be explained by the inability of SOD to reduce H_2_O_2_ formation. Curcumin also decreased the membrane lipid mobility coefficient in both cell lines. Notably, tetrahydrocurcumin did not affect ROS production or membrane mobility in either cell line [29]. Also, in HSG cells, curcumin and α-diisoeugenol exhibited similar IC_50_ values. However, at 10 µM, α-diisoeugenol induced a percentage of apoptotic cells comparable to the control, whereas curcumin caused a significant increase in apoptosis. In addition, curcumin promoted apoptosis in a dose-dependent manner up to 10 µM, with higher concentrations failing to further enhance cell death. A treatment duration of 5 h at 10 µM was required for significant apoptosis induction. Curcumin also generated ROS in HSG cells in a dose-dependent manner, whereas α-diisoeugenol had no effect. ROS production by curcumin was markedly potentiated in the presence of Cu(II) or Zn(II). Furthermore, in RAW cells, curcumin inhibited LPS-induced COX-2 expression, an effect not observed with α-diisoeugenol [143].

In nasopharyngeal carcinoma (NPC) cells, curcumin led to G2/M cell cycle arrest by downregulating CDK1 and cyclin B expression and induced apoptosis by increasing ROS production, upregulating BAX, and downregulating BCL-2, resulting in cytochrome c release and activation of caspase-9 and caspase-3 [144].

Similarly, a different study evaluating curcumin in OSCC showed it induced cell arrest at the G2/M phase but also apoptosis in vitro, while in vivo tumor growth was hindered [145].

In tongue squamous cell carcinoma (TSCC), curcumin reduced migration rate and upregulated protease-activated receptor-4, a pro-apoptotic protein, and decreased survivin expression, which induced apoptosis. Octamer-binding transcription factor 4 (OCT4) was also downregulated, suggesting that curcumin can suppress tumorigenesis [146].

In the SCC-4 oral cancer cell line, curcumin reduced cyclin B, CDK1 and cell division cycle (CDC) 25C levels, leading to cell arrest at the G2/M phase. Curcumin also decreased the expression of BCL-2, X-linked inhibitor of apoptosis protein (XIAP) and procaspases-8 and -9 while increasing cytochrome c release and caspase-3 activation, suggesting apoptosis induction. In addition to caspase-dependent apoptosis, curcumin triggered a caspase-independent mitochondrial apoptotic pathway, characterized by the upregulation and mitochondrial release of apoptosis-inducing factor (AIF) and endonuclease G (Endo G), which promoted the trafficking of activating transcription factor (ATF)-4 and GADD153. Moreover, curcumin increased intracellular Ca^2+^ release and upregulated endoplasmic reticulum (ER) stress-related proteins, including GRP78, protein kinase RNA-like endoplasmic reticulum kinase, inositol-requiring enzyme-1, ATF-6, and GADD153. Thus, it is suggested that curcumin promotes apoptosis in SCC-4 cells through both mitochondrial-dependent pathways and ER stress-mediated mechanisms [147]. Curcumin also triggered apoptosis in the H5 cell line by reducing BCL-2 expression and upregulating BAX, inducing caspase-9 activation. These effects are, at least in part, explained by the curcumin-induced reduction of STAT3 expression since STAT3 is a known transcription factor associated with BCL-2 expression [148]. Furthermore, curcumin was also found to upregulate ATF-3, leading to an increased apoptotic rate in the MDA-1986 cell line [149].

In a different study, increased expression of BCL-2-interacting killer and BCL-2-interacting mediator of cell death (BIM) was reported after curcumin treatment in the FaDu cell line, suggesting that curcumin induces apoptosis through the regulation of these proteins since it did not significantly alter the expression of other pro- and anti-apoptotic BCL-2 family members. Moreover, repression of EGFR, human epidermal growth factor receptor 3, Akt, STAT3, NF-κB and ERK1/2 activation and reduction of cyclin D1 and cyclin D2 expression were observed in both cell lines in the study [150].

Using HNSCC cell lines, curcumin also induced apoptosis by promoting the degradation of S-phase kinase-associated protein 2 (Skp2), leading to the upregulation of p27 and p21, which inhibit CDKs. It also led to decreased levels of BCL-2, XIAP, cIAP-1, cIAP-2 and MMP and upregulation of BAX and cytochrome c, resulting in activation of caspases-3 and -9 and PARP cleavage [151].

On the other hand, the antitumoral effects of curcumin in some HNSCC cell lines were attributed to its interference with the ataxia-telangiectasia mutated (ATM)-checkpoint kinase 2 (CHK2)-p53 pathway. However, most of the experiments were only conducted using the HEp-2 cell line [152]. Nonetheless, another study demonstrated that curcumin suppressed cell proliferation and migration and induced apoptosis by increasing sirtuin 1 activity, leading to p53 deacetylation and activation of the ATM/CHK2 signaling pathway. This activation resulted in PARP cleavage and the activation of caspases-3, -8, and -9. Inhibition of ATM phosphorylation almost completely abrogated curcumin-mediated cell viability suppression, confirming the central role of the ATM/CHK2 pathway in curcumin-induced apoptosis. Curcumin also reduced IκBα activity. In vivo, curcumin significantly decreased tumor growth, an effect that was partially reversed by ATM inhibition. Additionally, curcumin reduced the expression of VEGF and MMP-2, indicating suppression of angiogenesis and invasion [153].

Curcumin was also shown to promote the formation of nuclear aggregates of mutant p53 in several cell lines, including SCC-9 and AW13516, but not of wild-type p53. In AW13516 cells, curcumin significantly reduced clonogenic potential. It was also suggested that curcumin inhibits mutant p53 deubiquitination, promoting its nuclear aggregation and, ultimately, leading to cell death [154].

In the Cal27 cell line, curcumin reduced cell migration, induced cell cycle arrest at the S phase and increased apoptosis, leading to increased BAX and caspase-3 expression and reduced BCL-2. Additionally, through bioinformatic analysis, several potential target genes were found, including *TK1*, *TDRD3*, *TAGLN2*, *RNASEH2A*, *PDE2A*, *NCF2*, *MAP3K3*, *GPX3*, *GPD1L*, *GBP1*, *ENO1*, *CAT*, *ALDH6A1*, *AGPS* and *ACACB*. These genes are mostly involved in oxidation–reduction processes, ROS metabolic processes, hydrogen peroxide catabolic processes, oxidoreductase activity and the peroxisome-related pathway [155].

In the SCC-25 cell line, curcumin reduced cell viability and clonogenic ability; induced cleavage of PARP and caspases-3, -6, -7 and -9; degraded lamin A/C; released cytochrome c and AIF; translocated DFF40/CAD to the nucleus; downregulated BCL-2; upregulated BAX; and caused a gradual decrease in proteasome activity, promoting apoptosis. Decreased expression of CDK2, CDK4, cyclin D1 and cyclin D2 and increased levels of p27 were also observed after curcumin treatment [156].

Furthermore, curcumin inhibited the proliferation of Epstein–Barr virus (EBV)-positive HK1-EBV cells in a dose-dependent manner and induced cell-cycle arrest at the G2 phase. In parallel, curcumin promoted apoptosis, as evidenced by increased expression of p53, Fas, Fas ligand, and elevated levels of cleaved caspase-3 and PARP-1. Curcumin also reduced the half-life of EBV nuclear antigen 1 (EBNA1) following cycloheximide treatment compared with control cells. Notably, treatment with the proteasome inhibitor MG-132 increased EBNA1 expression both in the presence and absence of curcumin, indicating that curcumin promotes EBNA1 degradation through a proteasome-dependent ubiquitin pathway. Consistent with this mechanism, overexpression of EBNA1 attenuated the effects of curcumin on cell viability. Additionally, curcumin reduced the production of EBV progeny virions in a dose-dependent manner, suggesting inhibition of both latent and lytic EBV replication. Moreover, curcumin also significantly decreased the expression of the EBV immediate-early transcriptional activator Zebra in both induced and non-induced HK1-EBV cells [157].

Curcumin was also found to induce apoptosis in a cell line of TSCC fibroblasts but not in a normal HGF cell line. Apoptosis induction was not associated with ROS production since curcumin reduced it in both cell lines [158].

Curcuminoids reduced cell viability in the Detroit 562 cell line by inducing caspase-3 activation, and they also inhibited NF-κB activity. HONE-1 cells were included in the study; however, this cell line is likely a derivative of HeLa and another cell of unknown origin [122,159]. In the HNO97 cell line, curcuminoids also reduced cell viability while increasing apoptosis, DNA damage and cell arrest at G2/M [160].

DBA was also found to induce apoptosis in the HSC-4 and HSC-2 cell lines since PARP cleavage, caspase-3 activation and nuclear condensation were observed after treatment with DBA. DBA-mediated apoptosis induction was achieved by downregulation of Sp1, which leads to the activation of BAX [161]. Similarly, in YD-15 cells, DBA downregulated Sp1, leading to the induction of apoptosis by increased BIM expression and cleavage of BH3-interacting domain death agonist (BID), caspase-3 and PARP [162].

The structural analog of curcumin, CLEFMA, induced apoptosis in OSCC cell lines. CLEFMA led to the downregulation of cIAP-1 and increased the phosphorylation of ERK, JNK and p38, leading to upregulation of heme-oxygenase 1 (HO-1) and subsequently activating caspase-3, -8 and -9 [163]. Similarly, GO-Y078 led to upregulation of HO-1 by promoting the binding of AP-1 to DNA through the activation of the p38/JNK1/2 pathways, inducing activity of caspases and consequently apoptosis. HO-1 might also induce cell death through the promotion of ferroptosis, resulting in ER stress and PUMA upregulation. GO-Y078 also increased SMAC/DIABLO expression, which inhibits IAPs activity, also promoting the activity of caspases. Besides promotion of cell death, GO-Y078 also led to cell arrest at the G2/M phase. Additionally, GO-Y078 showed higher anticancer potential than DMC [164].

DMC was shown to repress OSCC cell proliferation by inducing cell arrest at the G2/M phase and by promoting apoptosis. The mechanistic explanation for this effect is that DMC decreased expression of cIAP-1 and XIAP while increasing expression of HO-1 and inducing activation of caspase-3, -8 and -9. DMC also activated p38 and JNK1/2, but only p38 silencing prevented DMC-induced HO-1 upregulation and activation of caspase-3, -8 and -9 [165]. Moreover, DMC was also found to induce apoptosis by enhancing the interaction between ubiquitin-specific peptidase 13 (USP13) and phosphatase and tensin homolog (PTEN). This interaction stabilizes PTEN, which in turn prevents Akt activation, leading to the repression of hexokinase 2 (HK2). HK2 promotes aerobic glycolysis, and thus, DMC promotes apoptosis by inhibiting glycolysis [166].

In oral cancer cells, FLLL32 promotes cell cycle arrest at the G2/M phase and apoptosis by inducing p38 activation, leading to HO-1 upregulation and activation of caspase-3, -8 and -9 [68]. In HNSCC cell lines, FLLL12 was more cytotoxic than curcumin. FLLL12 induced apoptosis by decreasing BCL-2 and BID expression, while it upregulated BIM. It also reduced transcription of EGFR and Akt while inhibiting their activation, but also of their downstream targets, including mTOR, S6, 4EBP1, FOXO1 and FOXO3. In vivo, FLLL12 also reduced tumor growth more effectively than curcumin [63].

HO-3867 was also found to promote apoptosis through the activation of the JNK1/2 pathway. Even though increased phosphorylation of ERK1/2, p38 and JNK1/2 was observed, only inhibition of JNK1/2 rescued the increased cleavage of caspase-3, -8 and -9 induced by HO-3867. The activation of JNK1/2 signaling probably leads to the decreased expression of cIAP-1, XIAP and surviving that is observed after HO-3867 administration, leading to cell death [167]. Similarly, L48H37 also increased cleavage of caspase-3, -8 and -9 and downregulated cIAP and XIAP through the activation of the JNK/p38 signaling pathway [168]. However, the curcumin analog EF24 was found to induce cleavage of caspase-3 and -9 by decreasing the activation of the MAPK/ERK pathway [169]. The MAPK/ERK pathway activation can lead to apoptosis, but it can also prevent it. This can be explained by the fact that ERK1/2, JNK and p38 regulate both pro- and anti-apoptotic BCL-2 family members. ERK1/2 usually promotes the downregulation of pro-apoptotic proteins and upregulation of anti-apoptotic proteins, while JNK activates pro-apoptotic proteins like BAX and p53 and represses anti-apoptotic ones such as BCL-2 and myeloid cell leukemia (MCL)-1. Nonetheless, their function depends on the context of their activation. For instance, ERK1/2 promotes apoptosis in response to cisplatin-induced DNA damage, while JNK attenuates cell death in response to ER stress [170].

In 7,12-dimethylbenzanthracene (DMBA)-induced hamster buccal pouch tumors, dietary turmeric led to decreased tumor growth, even in the continued presence of DMBA, by downregulating survivin, cyclin D1, COX-2, BCL-2 and proliferating cell nuclear antigen (PCNA), which are involved in DNA replication and cell cycle control, repressing MAPK activation and upregulating BAX, caspase-3, caspase-9 and cytochrome c [171].

Besides apoptosis, curcumin can also induce autophagy since its administration was found to increase autophagic vacuole formation. It seems that curcumin promotes autophagy by increasing ROS production, potentially through the activation of JNK and repression of NF-κB [172].

Curcumin, DMC and BDMC led to apoptosis activation in SAS cells since increased activity of caspase-3 and -9; increased expression of BAK, caspase-6 and AIF; and downregulation of caspase-7, BCL-2 and Apaf-1 were observed for all compounds. They also induced autophagy since formation of autophagosomes; decreased phosphorylation of EGFR, Akt, ERK1/2, JNK1/2, m-TOR and p38; and expression of PI3K, PKC and glycogen synthase kinase-3 (GSK-3)α/β were observed, along with increasing AMPKα1, Beclin 1, p62/SQSTM1 and LC3. Paradoxically, increased expression of BCL-2 and reduction of BAX and BID were associated with curcumin, DMC and BDMC treatment. However, BCL-2 has been shown to interact with Beclin-1 to suppress expression of BAX, BID and BAD during autophagy progression. Moreover, these compounds induce autophagy through ROS production [173].

PAC was also found to induce cell cycle arrest, apoptosis and autophagy in oral cancer cells. The effect of PAC on cell cycle and cell proliferation was due to the upregulation of p21, p27, p16 or p53 and Rb, which inhibit CDKs, and to the repression of cyclin D1 and c-Myc, which are oncogenes. Regarding apoptosis induction, PAC downregulates BCL-2 while it upregulates BAX and cytochrome c, leading to caspase-3 and -9 and PARP-1 cleavage and, consequently, apoptotic cell death. The effects on cell cycle and cell death promotion can be explained by PAC-mediated decreased phosphorylation of ERK1/2, p38, STAT and Akt, and reduced expression of NF-κB and β-catenin. However, PAC can also inhibit H2A.X protein expression, attenuating DNA damage. Autophagy induction was confirmed through the increased expression of the autophagy markers LC3B and p62. Nonetheless, PAC prevented ROS production by increasing glutathione (GSH) expression. It is suggested that high proliferative cells produce ROS at higher levels and that cancer cells adapt to PAC treatment by increasing their antioxidant status to optimize ROS-driven proliferation. Nonetheless, PAC also increased mitochondrial superoxide production and reduced mitochondrial membrane potential, resulting in the release of cytochrome c [174].

In Cal27 cisplatin-resistant cells, MTH-3 reduced cell proliferation and induced autophagy. MTH-3 induces autophagy by reducing PI3K/Akt/mTOR signaling. Active Akt phosphorylates transcription factor EB (TFEB), which is involved in the transcription of genes involved in autophagy and lysosomal biogenesis, preventing its translocation to the nucleus. MTH-3 repression of Akt increased TFEB activity, promoting autophagy. Moreover, PI3KC3 expression is also increased after treatment with MTH-3 due to repression of the PI3K/Akt/mTOR pathway, increasing Beclin-1 activity. EGFR was also downregulated, which is associated with autophagy induction. MTH-3-promoted autophagy results in apoptotic cell death, which is demonstrated by the increased caspase-3 and -9 cleavage, BAX and BAD expression, downregulation of BCL-2 and BCL-xL and reduction of mitochondrial membrane potential observed [81].

By influencing pro- and anti-apoptotic proteins, as well as autophagy-related pathways, curcumin can trigger selective tumor cell death while sparing normal cells, highlighting its potential as a multifaceted anticancer agent.

### 3.4. Migration and Invasion Regulation

Migration and invasion are highly associated with aggressiveness and poor prognosis in HNC [175]. Accordingly, curcumin and its analogs have been extensively investigated for their potential to inhibit these processes.

**Figure 3:**
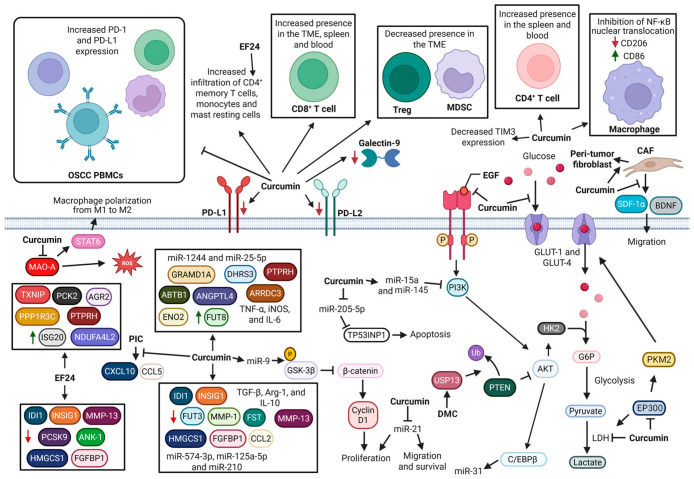
Curcumin and analogs’ effects on migration, immune system and metabolism of oral cancer cells. Curcumin increases the expression of several proteins and miRNAs involved in the regulation of metabolism, migration and the immune system, such as FUT8, PTPRH, and miR-1244, while it downregulates others, such as IDI1, MMP-13, Galectin-9, PD-L1 and miR-574-3p. Moreover, curcumin inhibits PIC promotion of CXCL10 and CCL5 expression, MAO-A repression of STAT6 and ROS production, EGF signaling, glucose uptake, EP300 inhibition of GLUT1 expression, CAF release of SDF-1α and BDNF, LDH prevention of lactate formation, miR-205-5p promotion of apoptosis, and miR-21 repression of proliferation, migration and survival. Curcumin also promotes miR-15a and miR-145 inhibition of PI3K and miR-9, leading to proliferation inhibition. Curcumin also increases presence of antitumor immune cells while reducing protumor immune cells in the TME. DMC also represses glycolysis by promoting USP13 activity, leading to PTEN deubiquitination and resulting in HK2 inhibition. EF24 increases the infiltration of CD4^+^ T cells in the TME, while it reduces the expression of proteins involved in proliferation, migration and metabolism, such as MMP-13, FGFBP1, HMGCS1, and increases the expression of others, like PTPRH, ISG20 and PCK2. The green arrow represent upregulation while the red arrows represent downregulation.

For instance, in OSCC cell lines, curcumin repressed cell invasion through the downregulation of MMP-2, MMP-9, urokinase-type plasminogen activator (uPA) and/or urokinase-type plasminogen activator receptor (uPAR) expression through the repression of hepatocyte growth factor signaling by preventing the activation of the c-Met/ERK pathway and by repressing ERK/MAPK and NF-κB activation (Figure 3). MMPs degrade collagen and other proteins in the extracellular matrix, while uPA is essential for the conversion of plasminogen to plasmin, which is also involved in the degradation of the extracellular matrix [16,176,177,178,179]. A similar study showed that curcumin reduced the expression of MMP-2 and MMP-9 but also Snail and Twist, transcription factors involved in the repression of E-cadherin transcription, through the induction of p53 expression. p53 seems to promote Snail and Twist degradation, leading to increased E-cadherin expression and EMT repression [180]. In SCC-1 and SCC-4 cells, curcumin reduced the expression of vimentin and Twist, leading to suppression of EMT, and concurrently induced apoptosis [181].

In TSCC cell lines, curcumin repressed cell proliferation, migration and invasion. Cell migration and invasion were repressed by the curcumin-mediated downregulation of MMP-10 [182]. Similarly, a different study also showed that curcumin reduced cell viability while also reducing cell invasion of 3D Myogel spheroids without affecting their diameter [183].

Moreover, curcumin was shown to decrease cell proliferation and increase apoptosis more effectively in cells with a mesenchymal-like phenotype. Low doses of curcumin reduced cell migration speed, and in 3D spheroids of lower and highly invasive OSCC, curcumin impaired their stability, while it did not affect non-tumor cells. Addition of curcumin reduced tumor growth in vivo, and less aggressive histological phenotypes were observed [184].

In Cal27 cells, curcumin significantly reduced cell migration and colony-forming ability. Additionally, curcumin modulated multiple signaling pathways involved in cell growth, survival, migration and invasion. It induced hyperphosphorylation of several phosphatases, including protein phosphatase 1 catalytic subunit beta (PPP1CB), which is involved in cell cycle regulation, protein tyrosine phosphatase receptor type K (PTPRK), protein tyrosine-protein phosphatase, non-receptor type (PTPN) 6 and PTPN11, while repressing phosphorylation of protein tyrosine phosphatase receptor type E (PTPRE). PTPRK negatively regulates STAT3 activation, whereas PTPRE promotes c-Src activation, which subsequently activates GRB2-associated binding protein 1 (GAB1), a mediator of cell growth and survival. PTPN6 and PTPN11 are also implicated in cell growth regulation, with PTPN6 acting as a tumor suppressor by inhibiting proliferation and promoting apoptosis. Curcumin additionally increased phosphorylation of several tyrosine kinases, including Abelson tyrosine-protein kinase 2 (ABL2), inositol polyphosphate phosphatase-like 1 (INPPL1), MAPK9, MAPK11, MAPK12, and ephrin receptor A2 (EPHA2), while decreasing phosphorylation of Fyn-related Src family kinase (FRK), pseudopodium-enriched atypical kinase 1 (PEAK1), tyrosine kinase non-receptor 2 (TNK2), the receptor tyrosine kinase AXL, and ERK1/2. MAPKs play key roles in regulating proliferation and apoptosis, whereas FRK, PEAK1, and TNK2 are associated with migration and invasion, and AXL is linked to angiogenesis. Furthermore, curcumin increased phosphorylation of caveolin-1 (CAV-1), whose overexpression is known to reduce integrin β3 levels, leading to repression of FAK activity and subsequent inhibition of cell migration. In parallel, curcumin decreased PI3K phosphorylation, further contributing to the suppression of invasion and migration [185]. Moreover, curcumin also led to higher phosphorylation of FAK; serine/threonine-protein kinase (STK) 17A, involved in apoptosis and proliferation; cortactin, which regulates cell shape and migration; and STAT1, while reducing EPHA2; calcium/calmodulin-dependent protein kinase kinase 2 (CaMKK2); p21-activated kinase 1 (PAK1), a regulator of cell survival, growth and motility; EGFR; STK10, involved in lymphocyte migration; acetyl-CoA carboxylase 1 (ACC1), which regulates fatty acid synthesis; 5′-AMP-activated protein kinase subunit beta-1 (PRKAB1), which controls cellular energy homeostasis; and MAPK1 and MAPK3 phosphorylation. In HNSCC cells, the inhibition of CaMKK2 reduced invasion and colony-forming ability through the repression of its target AMPKα activation [186].

In NPC, curcumin reduced NF-κB expression while upregulating E-cadherin, reducing cell migration. Similar results were observed in mice, but the cell line used to create mouse xenografts, HONE-1, is probably a derivative of HeLa and another cell of unknown origin [124,187].

In Tu686 and AMCHN-8 cells, curcumin reduced the expression of PCNA and upregulated p21, leading to an increased percentage of cells arrested at G0/G1. Curcumin also inhibited angiogenesis in the presence of HUVECs and repressed cell migration by downregulating the transcription factor E2F1. Reduced E2F1 expression subsequently led to decreased levels of filamin A (FLNA). Consistently, FLNA overexpression reversed the inhibitory effects of E2F1 suppression on angiogenesis and cell migration and was associated with increased PCNA expression and reduced p21 levels. In vivo curcumin treatment significantly reduced tumor growth and downregulated E2F1 and FLNA [188].

Curcumin exerted cytotoxic effects on HSC-3 cells in both a dose- and time-dependent manner while reducing glucose uptake, decreasing lactate dehydrogenase (LDH) activity, and lowering lactate production, indicating suppression of aerobic glycolysis. Curcumin induced cell-cycle arrest at the G0/G1 phase and downregulated the expression of hypoxia- and angiogenesis-related genes, such as STAT3, MMP-3, VEGF, and HIF-1α. Molecular docking analysis showed that of the genes evaluated, curcumin had the highest affinity with MMP-3 [189].

PAC was also found to suppress EMT and cell migration since it led to upregulation of E-cadherin and downregulation of vimentin [174].

In a multidrug-resistant OSCC cell line, mono-O-demethylcurcumin was found to inhibit proliferation and invasiveness more effectively than curcumin [83].

In nasopharyngeal cancer cell lines, L48H37 and EF24 were shown to reduce cell migration and invasion in the presence of TPA, a known promoter of cell invasion, by reducing MMP-9 expression through the inhibition of TPA-induced JNK activation. The combination of L48H37 or EF24 with a JNK inhibitor, JNK-IN-8, further decreased MMP-9 expression and activity and, consequently, cell migration [190,191].

Thus, the available evidence indicates that curcumin and its analogs appear to exert significant anti-invasive and anti-metastatic effects in HNSCC by targeting EMT, angiogenesis, and multiple oncogenic signaling pathways, highlighting their potential as promising therapeutic agents to prevent or attenuate tumor progression and aggressiveness.

### 3.5. Modulation of Lipid Metabolism

Reprogramming of lipid metabolism is a hallmark of cancer and contributes to enhancing proliferation, survival, and invasion. HNCs exhibit significant alterations in lipid metabolic pathways, making the targeting or reversal of these changes an attractive therapeutic strategy [192].

In this sense, curcumin has been shown to modulate genes involved in glycosylation and lipid metabolism. In SAS oral cancer cells, it upregulated FUT3, which is associated with favorable prognosis, and downregulated FUT8, which correlates with poor clinical outcomes, while reducing cell proliferation [193]. Similarly, in UM-SCC-1 and HSC-3 cells, curcumin inhibited cell viability, migration, glucose uptake, and lactate production, suggesting suppression of glycolysis through downregulation of EP300, involved in transcription regulation, which in turn leads to the reduction of glycolytic enzymes, such as pyruvate kinase M2, glucose transporter 1 (GLUT1) and LDHA [194].

In OSCC cells, curcumin and EF24 reduced cell proliferation, migration, invasion, and clonogenic capability while promoting apoptosis, with EF24 exhibiting greater potency, as reflected by lower IC_50_ values. Transcriptomic analysis showed that the seven most upregulated genes by curcumin were dehydrogenase/reductase 3, associated with lipid and retinoid metabolism; enolase 2, a key glycolytic enzyme that plays a role in energy metabolism; Gram domain containing 1a, which contributes to cholesterol homeostasis; protein tyrosine phosphatase, receptor H (PTPRH), a regulator of cell growth and motility; ankyrin repeat and BTB domain containing 1, implicated in growth inhibition; angiopoietin-like 4, which inactivates lipoprotein lipase and modulates lipid uptake in macrophages; and arrestin domain-containing protein 3, a known tumor suppressor, while the seven most downregulated genes were 3-hydroxy-3-methylglutaryl-CoA synthase 1 (HMGCS1), essential for the mevalonate pathway; isopentenyl-diphosphate isomerase 1 (IDI1), which also plays a role in the mevalonate pathway; insulin-induced gene 1 (INSIG1), which plays a role in lipid metabolism homeostasis; MMP-1, MMP-13, and fibroblast growth factor binding protein 1 (FGFBP1), which are associated with cell proliferation, differentiation and migration; and follistatin, inhibitor of the transforming growth factor-β (TGF-β) superfamily. On the other hand, the seven most upregulated genes by EF24 are thioredoxin-interacting protein (TXNIP), which plays a role in metabolism maintenance; phosphoenolpyruvate carboxykinase-2, which participates in gluconeogenesis; protein phosphatase 1 regulatory subunit 3C, a regulator of glycogen biosynthesis; NADH dehydrogenase (ubiquinone) 1 alpha subcomplex, 4-like 2, a component of Complex I of the mitochondrial respiratory chain; PTPRH, anterior gradient 2, involved in oxidative protein folding; and interferon-stimulated gene 20 kDa, an antiviral protein, while the seven most downregulated genes were HMGCS1; IDI1; INSIG1; FGFBP1; MMP-13; proprotein convertase subtilisin/kexin type 9, which promotes degradation of the low-density lipoprotein receptor; and ankyrin-1, involved in cell structure. Protein–protein interaction network analysis revealed that 3-hydroxy-3-methylglutaryl-CoA reductase (HMGCR), which plays a role in mevalonate pathway; squalene epoxidase, important for cholesterol homeostasis; INSIG1; HMGCS1; fatty acid synthase (FASN), involved in the biosynthesis of saturated fatty acids; and lanosterol 14α-demethylase (CYP51A1), required for cholesterol synthesis, were the most highly interacting proteins in the low-concentration curcumin group, whereas HMGCS1; FASN; INSIG1; ATP citrate lyase (ACLY), involved in lipid synthesis and metabolism; and CYP51A1 were dominant at higher concentrations. Similarly, EF24 exposure highlighted FASN; JUN, a proto-oncogene; ACLY; 7-dehydrocholesterol reductase, involved in cholesterol production; HMGCR; and FOS as major interaction nodes at lower doses, while FASN, HMGCR, CAV1, HMGCS1, INSIG1, and ACLY were identified as central nodes in the high-concentration EF24 group. Thus, the authors suggest that both curcumin and its analog EF24 may exert antitumor effects in OSCC cells through the downregulation of lipid metabolism. Furthermore, the KEGG pathway enrichment analysis demonstrated that the top-ranked pathways following curcumin treatment were metabolic pathways, the PI3K/Akt signaling pathway, and the MAPK signaling pathway, whereas the most significantly enriched pathways after EF24 treatment included the PI3K/Akt signaling pathway, the MAPK signaling pathway, and HPV infection [45].

Furthermore, DMC was found to reduce cell viability, induce the intrinsic apoptotic pathway with minimal cytotoxicity towards normal cells, and reduce glucose consumption and lactate production, thus promoting a metabolic shift from aerobic glycolysis toward oxidative phosphorylation in OSCC cells by downregulating HK2 expression and its mitochondrial localization. Mechanistically, DMC enhanced USP13-mediated deubiquitination of PTEN, leading to Akt pathway inactivation and subsequent downregulation of HK2. In addition, DMC treatment significantly inhibited tumor growth, reduced Akt activation and HK2 expression, and prolonged survival in vivo [166].

Overall, curcumin and curcumin-derived compounds exert antitumor effects through the modulation of multiple metabolic pathways, particularly glycolysis, lipid biosynthesis, and cholesterol metabolism. Their ability to target central metabolic regulators while simultaneously affecting oncogenic signaling pathways highlights metabolism-directed therapies as a promising strategy for the treatment of HNSCC and OSCC.

### 3.6. Epigenetic Modulation and microRNA Regulation

MicroRNAs (miRNAs) are key members of the non-coding RNA family that regulate multiple cellular processes, including growth and proliferation, cell cycle progression, differentiation, apoptosis, and tissue development. Dysregulation of miRNA expression in cancer cells can contribute to malignant phenotypes [80].

Curcumin has been shown to modulate the expression of miRNAs, and its effects in HNSCC will be discussed below. For instance, in the SCC-9 cell line, curcumin led to the inhibition of cell proliferation by upregulating miR-9. miR-9 upregulation led to increased expression of GSK-3β, phosphorylated GSK-3β and β-catenin inhibiting the Wnt/β-catenin pathway, leading to the reduction of cyclin D1 levels [195]. In OSCC, EGF was shown to increase Akt activation and to upregulate CCAAT/enhancer-binding protein (C/EBP) β, resulting in miR-31 upregulation, which is involved in EGFR-mediated carcinogenesis. Treatment with curcumin represses Akt activation and C/EBPβ upregulation, leading to the downregulation of miR-31 [196].

In the NPC cell line HK1, curcumin was shown to reduce miR-574-3p, miR-125a-5p and miR-210 and increase the expression of miR-1244 and miR-25-5p. Higher expression of miR-125a-5p was found to increase proliferation, migration and invasion; however, these experiments were conducted with the problematic cell line HONE-1 [122,197].

Curcumin upregulated miR-15a and miR-145 in laryngeal squamous cell carcinoma (LSCC) cell lines, leading to inhibition of cell proliferation and the PI3K/Akt/mTOR pathway and induction of apoptosis [198,199].

In the C6661 and the radioresistant C6661-IR cells, curcumin decreased miR-205-5p levels, resulting in increased expression of its target, the tumor suppressor TP53INP1, leading to reduced colony formation, cell viability and invasion, as evidenced by the upregulation of E-cadherin and downregulation of N-cadherin. Notably, overexpression of miR-205-5p or knockdown of TP53INP1 attenuated the effects of curcumin in C666-1-IR cells, suggesting that curcumin exerts its antitumor activity, at least in part, through upregulation of TP53INP1 [200].

In CSCs isolated from both HPV-positive and HPV-negative oral cancer cell lines, curcumin inhibited cell proliferation and orosphere formation and significantly reduced miR-21 expression. Nonetheless, these effects were slightly more pronounced in the HPV-positive CSCs. Orospheres derived from HPV-positive CSCs were less compact and less spherical, which may partly explain the greater sensitivity of these cells to curcumin-mediated inhibition of orosphere formation. Since miR-21 targets multiple tumor suppressor genes involved in cell proliferation, invasion, and survival, the observed reduction in CSC viability and proliferation may be partially attributed to curcumin-induced miR-21 downregulation [201].

In summary, curcumin has been found to regulate several miRNAs, further supporting the potential of curcumin as a multitargeted therapeutic agent capable of reprogramming epigenetic and post-transcriptional regulatory mechanisms in HNSCC.

### 3.7. Tumor Immune Microenvironment Modulation

Cancer cells can evade the immune system by altering chemoattractant pathways, which prevents the infiltration of immune cells in the TME, but also by hindering the antigen-presentation machinery to avoid being detected [202]. Oral cancer cells are no exception, and in this section, the effects of curcumin on the modulation of immune responses and tumor immune microenvironment (TIME) of OSCCs will be discussed.

For instance, peripheral blood mononuclear cells from OSCC patients have been shown to overexpress PD-1 and PD-L1, which are associated with immune system suppression. The addition of curcumin decreased both PD-1 and PD-L1 expression and might be a promising therapeutic option to reactivate immune responses [203]. Moreover, in TSCC, curcumin led to the reduction of cell proliferation but also to reduced expression of PD-L1 and phosphorylated STAT3, both in vitro and in vivo. Repression of PD-L1 expression and STAT3 activation led to increased presence of CD8^+^ T cells in the TME and reduced levels of regulatory T cells (Tregs) and myeloid-derived suppressor cells (MDSCs) [204].

In HNSCC cells, curcumin decreased the expression of proteins involved in the suppression of immune responses, such as PD-L1, PD-L2 and Galectin-9, which affects proliferation and invasion, while inhibiting STAT3 phosphorylation both in vitro and in vivo. When co-cultured with CD8^+^ T cells, curcumin showed higher cytotoxic effect, whereas in vivo, it increased the presence of CD4^+^ and CD8^+^ T cells in the spleen and blood and reduced CD4^+^ and CD8^+^ T cells expressing PD-1 or TIM3 and Tregs. IFN-γ and granzyme B, which are produced by cytotoxic T cells, were also increased after treatment with curcumin [205].

Curcumin was also found to significantly reduce C-C motif chemokine ligand (CCL) 22 protein levels when administered alone or in combination with the TLR3 ligand poly(I:C) (PIC) in ex vivo tissues. In macrophage cultures, PIC increased CCL5 and CXC motif chemokine ligand (CXCL) 10 expression; however, co-treatment with curcumin abolished the induction of CCL5 and rendered the increase in CXCL10 non-significant compared with the control. In the supernatants from curcumin-treated cancer specimens, either alone or combined with PIC, reduced Treg migration compared with control and PIC-alone treatments was observed, whereas in macrophage-derived supernatants, curcumin alone or combined with PIC reduced Treg migration only relative to PIC alone. Additionally, curcumin inhibited NF-κB nuclear translocation in macrophages and was shown to suppress NF-κB activity more effectively than the NF-κB inhibitors BAY 11-7082 and CAPE [181].

Curcumin has also been shown to regulate the TIME by modulating tumor-associated macrophages. For instance, HNSCC cells were found to promote macrophage polarization toward the M2 phenotype via CCL2 secretion, establishing a positive feedback loop in which M2-derived EGF enhances tumor invasiveness and further CCL2 expression. Curcumin treatment disrupted this axis by reducing CCL2 mRNA and protein levels in co-cultured Cal27 and THP1 cells, decreasing cortactin/F-actin and MMP-14/F-actin colocalization and ultimately suppressing Cal27 cell migration and invasion [206]. Moreover, the addition of curcumin led to reduced expression of anti-inflammatory cytokines such as TGF-β, Arg-1, and IL-10 while increasing proinflammatory ones, including TNF-α, iNOS, and IL-6. Curcumin also decreased expression of the M2 macrophage marker CD206 and increased the expression of CD86, a M1 macrophage marker, on the surface of Cal27 cells. M1 macrophages are pro-inflammatory and associated with antitumoral properties, while the anti-inflammatory M2 macrophages are associated with tumor growth and metastasis promotion. The increased levels of M1 macrophages and decreased levels of M2 macrophages in the TME prevented cell invasion and migration. The reprograming of macrophages from M2 to M1 was achieved through the inhibition of monoamine oxidase A (MAO-A). MAO-A induces ROS production and activation of the STAT6 pathway, which promotes the polarization of macrophages to an M2 phenotype [207].

Additionally, curcumin can also interfere in the interaction of cancer-associated fibroblasts (CAFs) and oral cancer cells. CAFs have been reported to induce EMT of OSCC cells by releasing stroma-derived factor 1α (SDF-1α) or brain-derived neurotrophic factor (BDNF), while treatment with curcumin decreases their gene expression in co-cultures of fibroblast and SCC-25 to levels observed in the control with only fibroblasts. This is potentially achieved by curcumin-induced reduction of integrin alpha (ITGA) 5 expression and the nuclear translocation of NF-kBα in fibroblasts. Nonetheless, curcumin also reduced the expression of ERK1/2 and NF-kBα in cancer cells, increasing E-cadherin expression and reducing EMT [208]. Curcumin can also revert CAF phenotype to a peri-tumor fibroblast phenotype since reduced expression of alpha-smooth muscle actin (α-SMA), a CAF-associated marker; and inhibition of CAF-mediated secretion of TGF-β1, MMP-2, and SDF-1, cytokines that promote carcinogenesis, were observed after its administration. In this way, curcumin repressed the proliferation of the TSCC cell line Cal27 promoted by CAFs. Similar effects were observed in vivo, where the stimulation of carcinogenesis promoted by CAFs was reduced [209].

Moreover, an immunocyte signature gene analysis showed that the curcumin-treated group had increased infiltration of CD4^+^ memory T cells, monocytes and mast resting cells, while EF24 led only to increased infiltration of CD4^+^ memory T cells [45].

In summary, the evidence suggests that curcumin exerts immunomodulatory effects in HNSCC by reshaping the TIME through downregulation of immune checkpoint molecules, enhancement of cytotoxic T-cell activity, reduction of immunosuppressive populations, reprogramming of tumor-associated macrophages from a pro-tumoral M2 phenotype toward an antitumoral M1 phenotype and interfering with CAF-mediated EMT and carcinogenic signaling, reinforcing its potential as an adjuvant immunomodulatory agent.

## 4. Role of Curcumin in Chemoprevention

Given the strong association of HNSCC with risk factors such as tobacco use, alcohol consumption, gastric reflux, and areca nut chewing, curcumin and its analogs have been extensively investigated as potential chemopreventive agents [3].

For instance, pretreatment of human laryngeal and hypopharyngeal epithelial cells with curcumin prior to exposure to pepsin, a gastric enzyme with pro-carcinogenic activity in the larynx and pharynx, significantly reduced pepsin substrate hydrolysis, cell proliferation, and the proportion of cells arrested in the S phase. Curcumin pretreatment also increased the expression of genes involved in cell cycle regulation and DNA damage repair (*BRCA1*, *CCNE1*, *E2F1*, *CDC25*, and *ATM*), apoptosis (*BAD*, *TNFRSF1A*, *BCL2L1*, *BCL2*, and *BAX*), signal transduction (*ERBB2*, *PIK3R1*, *AKT1*, and *SNCG*), cell adhesion (*SYK* and *ITGA1*), and angiogenesis (*FGFR2*, *COL18A1*, *TGFβRI*, *TGFβ1*, and *ANGPT2*), while reducing the expression of *ETS2*, compared with pepsin treatment alone. Additionally, in curcumin-pretreated cells, pepsin localization was no longer confined to intracellular vesicles but was frequently dispersed throughout the cytosol [210].

Acidic bile reflux can activate NF-κB in normal human hypopharyngeal cells, leading to the upregulation of anti-apoptotic and oncogenic genes and thereby promoting carcinogenesis.

In human hypopharyngeal primary cells, curcumin inhibits acidic-bile-induced NF-κB and STAT3 nuclear translocation; IκBα activation; and BCL-2, p65, TNF-α, STAT3, WNT5a, ΔNp63, c-REL, EGFR and IL-6 expression, with similar results in vivo for curcumin topical application before, after or in combination with acidic bile, while also reducing Ki67 and COX-2 [211,212].

Moreover, mTOR was increased while AKT1 decreased only after or in combination with acidic bile. These results suggest that curcumin can prevent the bile reflux-related early carcinogenic events in the hypopharynx [212]. Nonetheless, curcumin reduced cell viability in the control, neutral bile-treated cells and acidic bile-treated cells with similar cytotoxicity [211].

Surprisingly, in OSCC, curcumin induced the activation and nuclear translocation of the aryl hydrocarbon receptor (AhR), leading to the formation of transcriptionally active AhR-ARNT complexes. AhR plays a role in tobacco-related carcinogenesis since it is activated by the tobacco-associated carcinogen benzo(a)pyrene. Curcumin also increased the expression and activity of cytochrome P-450 (CYP) 1A1 and CYP1B1, which are involved in the bioactivation of (-)-benzo(a)pyrene-7R-trans-7,8-dihydrodiodiol. However, curcumin also inhibits this bioactivation, potentially by acting as a competitive substrate during CYP1A1 metabolism, which prevents benzo(a)pyrene-induced carcinogenesis [213]. In the SCC-9 cell line, curcumin was also found to repress CYP1B1 activation after co-treatment with benzo(a)pyrene, further corroborating the preventive role of curcumin in tobacco-related carcinogenesis [214].

Consumption of smokeless tobacco is also associated with increased risk of developing oral cancer. For instance, smokeless tobacco extract increases expression and activation of NF-κB and COX-2. In both oral premalignant and oral cancer cells, curcumin was found to prevent the nuclear translocation of NF-κB and inhibit COX-2 upregulation induced by smokeless tobacco extract while inducing apoptosis [119].

Furthermore, prior incubation with curcumin was shown to prevent DNA damage, including damage to the EGFR gene, in mini-organ cultures of the oropharynx exposed to tobacco smoke condensate [215]. Similarly, in oropharyngeal mucosa cultures, curcumin significantly reduced cigarette smoke condensate-induced DNA damage. However, addition of epigallocatechin-3-gallate (EGCG) to curcumin did not enhance this effect [216]. Moreover, the administration of curcumin C3 complex, which contains curcumin, DMC and BDMC, led to reduction of oral lesions and cell proliferation. Pretreatment with curcumin C3 complex in vitro attenuated fibroblast growth factor 2 (FGF2)-induced proliferation [217].

Areca quid chewing, like consumption of smokeless tobacco, increases the risk of developing oral cancer. HSP70 overexpression was found in Areca quid chewing-associated OSCC. Furthermore, non-metastatic and poorly differentiated OSCC groups showed higher levels of HSP70 expression than metastatic and well-differentiated ones. It is suggested that Areca quid chewing induces oxidative stress and releases copper, which are both associated with HSP70 upregulation. Arecoline is the main component of Areca quid, and it contributes to oral carcinogenesis. In vitro, addition of arecoline to the GNM cell line promoted HSP70 expression, suggesting that HSP70 expression may contribute to OSCC pathogenesis. Nonetheless, administration of curcumin repressed the arecoline-induced HSP70 upregulation, potentially through the inhibition of the AP-1 pathway [218].

In HNSCC, curcumin prevented the activation of the Akt/mTOR pathway induced by nicotine in vitro. The inhibition of Akt/mTOR signaling led to inhibition of cell proliferation but also of migration and invasion through the downregulation of pS6, which in turn led to reduced expression of MMP-9. Similar results were observed in vivo using a 4-NQO carcinogenic model, with curcumin also reducing oral carcinogenesis during both initiation and post-initiation phases [219,220]. 4-NQO is a synthetic carcinogen that mimics chronic tobacco consumption effects and is usually used to induce oral tumor formation in vivo.

After 12 weeks, administration of curcumin at 100mg/Kg attenuated dysplasia and atypia of tongue epithelium and reduced SOCs3, CDH2, vimentin and Twist expression, upregulating SOCs1 and STAT3 compared to mice only treated with 4-NQO [221]. In a different study, curcumin was shown to reduce total malondialdehyde (MDA) levels in tissues during the initiation phase and to decrease both MDA and GSH levels in serum during the initiation and post-initiation phases. The reduction in MDA levels highlights the antioxidant properties of curcumin, whereas the decrease in GSH may be explained by its conjugation with GSH to form curcumin–glutathione complexes, potentially a more potent product than curcumin alone. Additionally, curcumin significantly reduced dysplasia scores at both the initiation and post-initiation stages [222].

In hamsters with DMBA-induced buccal pouch tumor, turmeric or curcumin addition to the diet or local application did not increase survivability but led to lower tumor burden and DNA adducts [223]. In a different study, 80 mg/Kg body weight of curcumin prevented oral carcinogenesis in hamsters treated with DMBA while restoring the expression of antioxidants and detoxification agents such as vitamins E and C; reducing GSH, SOD, catalase, glutathione peroxidase and glutathione reductase; and reducing lipid peroxidation [224]. Another study exploring a DMBA-induced oral carcinogenesis model also confirmed the chemopreventive effect of curcumin. The effect was attributed to the curcumin reduction of DMBA-induced VEGF expression [225].

Furthermore, curcumin was also found to inhibit oral carcinogenesis in vitro and in vivo by activating p38, which in turn promotes C/EBPα-mediated transcription of insulin-like growth factor binding protein-5 (IGFBP-5) [226].

A curcumin gum formulation tested in healthy adults led to a significant reduction in TNF-α and CXCL1 levels. High expression of CXCL1 is associated with carcinogenesis by promoting angiogenesis and cell invasion, while TNF-α upregulation leads to aberrant NF-κB signaling, promoting the expression of proinflammatory proteins, which can induce transformation of normal cells into malignant cells [227].

Curcumin was also evaluated in NOM9 cells, a normal oral epithelial cell line; NOM9-CT cells, an immortalized oral mucosal epithelial cell line; MSK-Leuk1 cells derived from oral leukoplakia; and the oral cancer cell lines UMSCC22B and SCC-4. It was found that curcumin decreased cell growth more effectively in the immortalized, leukoplakia and cancer cells than in the normal cells. Moreover, it repressed cap-dependent translation by inhibiting the activation of 4E-BP1, eIF4G, eIF4B and Mnk1 and also downregulating eIF4E and Mnk1, but not in the normal cell line [228].

Dysregulation of arachidonic acid metabolism, particularly the 5-lipoxygenase (5-LOX) pathway, has been implicated in oral carcinogenesis and represents a potential target for cancer prevention. Accordingly, several compounds were evaluated for their inhibitory activity against 5-LOX. However, among the tested agents, curcumin exhibited the lowest theoretical activity index, indicating that it was the least effective inhibitor of 5-LOX, and thus, it was not further examined [229]. Moreover, a daily dose of curcumin at 80 mg/kg body weight in mice was not effective in preventing the development of oral cancer [230].

Overall, these findings suggest that curcumin is, for the most part, effective in preventing carcinogenesis by targeting multiple mechanisms, including suppression of chronic inflammation, oxidative stress, carcinogen activation, DNA damage and angiogenesis. Even though a study in mice found that curcumin could not prevent oral cancer development, it is possible that the dose used was not sufficient. Nonetheless, in vitro and in vivo models support curcumin as a promising candidate for the prevention of HNSCC development and progression in individuals exposed to established carcinogenic risk factors.

## 5. Role of Curcumin in Oral Potentially Malignant Disorders (OPMDs)

OPMDs are oral mucosal abnormalities that carry a statistically increased risk of progression to oral cancer and comprise leukoplakia, proliferative verrucous leukoplakia, erythroplakia, oral lichen planus (OLP), oral submucous fibrosis (OSMF), actinic cheilitis, palatal lesions of reverse cigar smoking, discoid lupus erythematosus, dyskeratosis congenita, oral lichenoid lesions, and oral graft versus host disease [231]. In this sense, several compounds have been explored in the treatment of these disorders, including curcumin.

For instance, curcumin in Orabase was evaluated in patients with oral graft-versus-host disease, and it led to reduced pain and disorder severity. This treatment option showed similar effects to triamcinolone, a glucocorticoid Orabase, and can be used in patients with oral graft-versus-host disease [232].

In patients with OLP, oral leukoplakia, and OSMF, curcumin treatment significantly reduced MDA and 8-hydroxydeoxyguanosine levels while increasing vitamin C and vitamin E concentrations in both serum and saliva. These changes were statistically significant following clinical cure and were accompanied by significant reductions in pain intensity and lesion size across all premalignant lesions, suggesting that curcumin enhances antioxidant status while attenuating lipid peroxidation and DNA damage [233]. Additionally, curcumin treatment led to improved mouth opening in patients with OSMF [234]. In patients with OSMF, OLP or speckled leukoplakia, treatment with Turmix tablet combined with local application of turmeric and honey significantly reduced burning sensation [235].

Bioinformatic analysis identified several proteins with high predicted binding affinity to curcumin in the context of OSMF, including MMP-9, p53, MYC, IL-6, TNF, β-catenin, HIF-1α, albumin, JUN, and EGFR. These interactions suggest that curcumin may exert its therapeutic effects in OSMF by modulating these proteins and their pathways [236].

The activation of the TGF-β signaling pathway is considered a central mechanism driving excessive collagen deposition in OSMF. In this sense, several FDA-approved drugs were assessed for their affinity towards TGFβRI and TGFβRII, and the molecular docking analyses demonstrated that curcumin, curcumin pyrazole, and DMC are the compounds with the strongest binding affinities and the highest docking scores. Among these compounds, curcumin showed favorable pharmacokinetic properties, including high gastrointestinal absorption and strong potential as a lead compound. In addition, curcumin displayed superior synthetic accessibility compared with curcumin pyrazole and DMC. Further target prediction analysis revealed that curcumin, curcumin pyrazole, and DMC are highly conserved toward enzymes and cytokine-related targets involved in cell growth, proliferation, and apoptosis. All three compounds were predicted to interact with conserved domains of cytokine-related receptors, including TLR and IL-1 receptors, which belong to the same cytokine receptor superfamily as TGF-β receptors. Moreover, toxicity profiling indicated that curcumin exhibits a more favorable safety profile than curcumin pyrazole and DMC. Thus, curcumin was considered by the authors as the most suitable lead candidate with promising potential for OSMF treatment [108]. Myofibroblasts also play a key role in the development of OSMF, and curcumin was shown to inhibit their proliferation more effectively than fibroblasts. This effect was accompanied by induction of apoptosis through downregulation of BCL-2 and upregulation of BAX. Additionally, curcumin reduced type I and type III collagen expression in a time- and dose-dependent manner [237].

Human oral mucosal fibroblasts (HOFs) exposed to arecoline, an OSMF cell model, exhibited increased cell viability, migration, and invasion compared to normal HOFs, all of which were significantly suppressed by curcumin treatment. Curcumin also promoted an epithelial phenotype, as evidenced by increased E-cadherin expression and reduced levels of N-cadherin and vimentin. In addition, curcumin attenuated activation of the TGF-β/SMAD signaling pathway by decreasing TGF-β1 expression and reducing SMAD2 and SMAD3 phosphorylation. Mechanistically, these effects were at least partially mediated by curcumin-induced downregulation of the long non-coding RNA XIST and concomitant upregulation of miR-25-3p, as inhibition of miR-25-3p or overexpression of XIST partially reversed the effects of curcumin [238]. In a different study, curcumin markedly reversed the effects of arecoline by significantly reducing cell viability and migration and downregulating collagen type I alpha 1 chain (COL1A1), α-SMA, IL-1β and IL-6 expression while simultaneously promoting apoptosis. These effects were mediated through curcumin-induced inhibition of HIF-1α, which led to decreased expression of its downstream target latent-transforming growth factor β-binding protein 2 (LTBP2). This reduction in LTBP2 resulted in attenuated phosphorylation of IκB-α and p65, thereby suppressing NF-κB signaling [239].

Arecoline has been shown to upregulate connective tissue growth factor (CTGF), a key mediator that might promote fibrotic activity and contribute to the pathogenesis of OSMF. In arecoline-treated buccal mucosal fibroblasts, curcumin significantly reduced CTGF expression [240]. These fibroblasts also present enhanced cell migration, increased expression of COL1A1, α-SMA, FOS-like antigen 1 (FOSL1), MAPK8, and elevated SMAD2 phosphorylation. Curcumin treatment suppressed FOSL1 expression and, consequently, downregulated its downstream target MAPK8. This modulation led to reduced cell migration, decreased SMAD2 phosphorylation, and lower expression of COL1A1 and α-SMA, thereby inhibiting arecoline-induced fibroblast activation [241].

p53, TGF-β and iNOS are associated with malignant transformation and are overexpressed in OSMF, suggesting their involvement in OSMF pathophysiology. In this sense, the effect of curcumin on the expression of p53, TGF-β and iNOS was investigated in patients with OSMF. Immunohistochemical analysis of patient samples collected before and after curcumin treatment revealed reduced expression of these markers following therapy, although these changes were not statistically significant [242]. Nonetheless, curcumin led to better clinical efficacy and improved mouth opening, tongue protrusion, cheek flexibility, clinical staging and histopathological grading, reduced swallowing difficulty and the percentage of patients with shrunken uvula, significantly reduced fibrosis and blanching in the oral mucosa with the exception of blanching in the floor of the mouth, reduced burning sensation and mucosal lesion area, increased IFN-γ and reduced TGF-β1 and TNF-α levels compared with the control group [243,244,245]. Moreover, both topical curcumin gel and buccal mucoadhesive patches showed similar effects: reducing burning sensation and LDH levels and improving mouth opening, tongue protrusion and cheek flexibility [246]. Curcumin and turmeric oil as chemopreventive agents in OSMF also produced comparable improvements in burning sensation, tolerance to spicy food, mouth opening, and histopathological grading [247].

Patients with OSMF were treated with curcumin tablets, curcumin capsules, or curcumin oil, with multivitamin tablets used as the control. All curcumin-based treatments led to improvements in burning sensation, tongue protrusion, and mouth opening compared with the control group. Among the treatment modalities, curcumin oil showed greater reduction in burning sensation, while curcumin capsules were associated with better improvements in mouth opening and tongue protrusion; however, these differences were not statistically significant [248]. A comparative study evaluating curcumin versus topical triamcinolone acetonamide, a corticosteroid, for the treatment of OSMF demonstrated that both therapies effectively improved mouth opening and reduced burning sensation. Although no statistically significant differences were observed between the two groups, curcumin treatment was associated with slightly better clinical improvement [249]. Nonetheless, a different study showed that curcumin lozenges significantly reduced burning sensation but did not improve mouth opening. In addition, the corticosteroid clobetasol showed superior clinical efficacy compared to curcumin [250].

Two separate studies reported that curcumin significantly reduced burning sensation and improved mouth opening; however, lycopene, a carotenoid, demonstrated superior clinical outcomes [251,252]. In other trials, both lycopene and curcumin produced similar overall effects in OSMF management. Lycopene showed slightly greater improvements in mouth opening and reduction of fibrous bands, whereas curcumin was more effective in alleviating burning sensation and blanching of the buccal mucosa; however, none of these differences reached statistical significance. Notably, both treatments improved cheek flexibility and reduced submucosal thickness, with lycopene showing a significantly higher reduction than curcumin [253,254].

Another study compared systemic curcumin combined with topical administration to an antioxidant formulation containing α-lipoic acid, β-carotene, copper, lycopene, selenium and zinc sulfate as active ingredients, or to systemic curcumin alone. While all treatment regimens improved mouth opening, burning sensation, and tongue protrusion, the combined systemic and topical curcumin approach produced the most pronounced clinical benefits [255].

A separate study evaluating curcumin delivered as lozenges in OSMF reported improvements in mouth opening and tongue protrusion, along with reduced lesion-associated burning sensation. When compared with intralesional dexamethasone and hyaluronidase administration, curcumin lozenges demonstrated superior clinical improvement [256]. However, Turmix, a tablet containing 300 mg of *C. longa* and 5 mg of piperine, showed inferior outcomes compared with intralesional dexamethasone and hyaluronidase in terms of interincisal distance and tongue protrusion, while only demonstrating a better effect on burning [257].

The comparison of curcumin and turmeric oil as chemopreventive agents in OSMF showed that both treatments produced comparable improvements in burning sensation, tolerance to spicy food, mouth opening, and histopathological grading [247].

Turmeric alone was found to significantly reduce burning sensation in patients with OSMF but did not produce a notable improvement in mouth opening [258]. Treatment with turmeric combined with cessation of betel nut habit resulted in the greatest reduction in MDA levels, ulceration, burning sensation, and fibrous band tightness, as well as the greatest increase in interincisal opening, compared with both the group receiving turmeric while continuing the betel nut habit and the group that discontinued the habit without turmeric treatment [259].

In patients with OSMF, treatment with 3 g/day of turmeric alone, 600 mg of turmeric oil combined with 3 g/day of turmeric, or 600 mg of turmeric oleoresin combined with 3 g/day of turmeric resulted in a reduction in micronuclei frequency in exfoliated buccal mucosal cells. Among these interventions, the combination containing turmeric oleoresin demonstrated the greatest efficacy. Additionally, all treatment regimens decreased the incidence of micronuclei in circulating lymphocytes, both in the presence and absence of benzo(a)pyrene, with no statistically significant differences observed between the treatment groups [260].

*Emblica officinalis* and *Curcuma longa* were also evaluated for the management of OSMF, and both treatments resulted in improvements in mouth opening, burning sensation, and reduction of fibrous bands. Notably, *Emblica officinalis* was more effective in alleviating burning sensation and enhancing mouth opening, whereas *Curcuma longa* showed greater efficacy in reducing posterior fibrous bands. Neither treatment produced significant improvement in mucosal blanching or tongue protrusion [261].

In OLP patients, curcumin capsules significantly reduced pain and improved the clinical appearance of oral lesions, with 50% of the patients reporting decreased pain and 10% achieving complete resolution. Moreover, 80% of patients showed improvement in the clinical appearance of their lesions [262]. Furthermore, curcumin achieved complete remission of pain in nine patients (36%), but only one patient showed complete remission regarding clinical response, while six patients (24%) showed no response for either variable evaluated. When compared with triamcinolone, no statistically significant differences were observed [263]. Similarly, in a different study, both 0.1% triamcinolone and 1% curcumin oral paste reduced burning sensation and improved appearance scores, with no statistically significant difference between the two treatments [264]. Furthermore, both 1% curcumin gel and triamcinolone acetonide were found to be effective in reducing lesion size and extent, as well as alleviating burning sensation, erythema, and ulceration. Nonetheless, triamcinolone acetonide showed better and early healing regarding erythema and ulceration [265]. Similarly, a study comparing 1% curcumin gel with triamcinolone acetonide found that both treatments reduced burning sensation, erythema, and ulceration, although triamcinolone was more effective [266]. Treatment with 1% curcumin gel, triamcinolone acetonide, or their combination resulted in reduced pain, disease severity, and IL-6 levels in OLP patients. The only statistically significant difference between groups at the end of treatment was in the Malhotra score, which assesses disease severity, with curcumin alone showing a smaller improvement compared to triamcinolone acetonide or the combination [267]. However, a different study showed that topical curcumin led to a greater erythema reduction than the 0.1% triamcinolone acetonide group. Nevertheless, both groups showed significant reduction in pain, erythema, and ulceration [268]. Moreover, a case report study showed that curcumin was effective in treating OLP and preventing recurrence in a patient previously treated with topical steroid [269].

In addition, curcumin lozenges demonstrated comparable efficacy to 0.1% triamcinolone acetonide paste in reducing pain severity after two weeks of daily use. In the curcumin group, five of 10 patients achieved complete symptom resolution, compared with four of 10 in the triamcinolone group. Additionally, three and two patients in the curcumin and triamcinolone groups, respectively, showed a very good response, while two and four patients demonstrated a good response. These findings suggest that curcumin may be a viable treatment option for OLP, offering effective symptom relief with minimal adverse effects [270].

A study comparing nano-curcumin with the corticosteroid prednisolone found no significant difference between the treatments, even though both reduced pain intensity, burning sensation, and lesion size [271]. Moreover, mucoadhesive pastes containing curcumin decreased lesion sizes, severity index and pain index in patients with OLP. However, no difference was found compared to the use of local corticosteroids [272]. Turmeric was also found to improve clinical symptoms such as burning sensation, redness and ulceration in OLP patients [273].

High-dose curcuminoids (6000 mg/day), after two weeks of treatment, were found to improve erythema, modified oral mucositis index (MOMI) and numerical rating scale (NRS). Compared with placebo, it showed improvement regarding percentage change of erythema and total MOMI score while showing the highest proportion of patients showing improvement in NRS and total MOMI score [274].

A follow-up study examined patients with OLP who had previously been enrolled in two clinical trials evaluating curcuminoid therapy, one using a daily dose of 2000 mg and the other 6000 mg. Following completion of the trials, patients continued curcuminoid supplementation on an over-the-counter basis. In the lower-dose cohort, the mean daily intake was 2137.5 mg over an average period of 30 months, whereas patients in the higher-dose cohort received a mean daily dose of 5058 mg for an average duration of 9.6 months. Improvement in symptoms was reported by 56% of patients in the lower-dose group and 63% in the higher-dose group. Adverse events were infrequent and dose-related: two patients (11%) in the lower-dose cohort experienced diarrhea, while six patients (32%) in the higher-dose cohort reported side effects, mainly abdominal discomfort and diarrhea. The symptoms in the higher-dose cohort were mitigated in four patients following dose reduction. Overall, across both cohorts, 60% of patients reported symptomatic improvement with curcuminoid therapy, 35% were uncertain about its effectiveness, and 5% reported no clinical benefit [275].

Treatment of patients with erythroplakia using a curcumin solid lipid nanoparticle–loaded mucoadhesive gel resulted in greater pain and lesion size reduction compared with a conventional curcumin mucoadhesive gel [276].

Oral leukoplakia patients treated with a 400 mg curcumin capsule exhibited a significant reduction in lesion size. Among the 30 patients evaluated, five achieved complete remission, 16 showed a partial response, and nine had stable disease [277]. The therapeutic effects of nanocurcumin were also evaluated in patients with oral leukoplakia, revealing significant reductions in lesion size, lesion number, and disease stage, along with an increase in SOD levels. Notably, a positive association was observed between post-treatment elevations in serum SOD and disease downstaging, indicating that increased SOD levels are predictive of treatment response [278]. Curcumin lozenges have also been evaluated for the treatment of oral leukoplakia and were found to reduce lesion size. In addition, histopathological analysis revealed overall improvement in both architectural and cytological features, supporting the potential use of curcumin in the management of mild dysplasia [279]. A study assessing curcumin and lycopene, administered either as monotherapies or in combination, for the treatment of oral leukoplakia found that the combined regimen produced the most favorable outcomes, with complete resolution observed in 72% of patients. In comparison, cure rates of 63% and 51% were reported for lycopene and curcumin alone, respectively. However, no statistically significant differences were observed between the lycopene monotherapy group and the combination group, nor between the lycopene and curcumin monotherapy groups [280].

A phase I trial evaluating curcumin as a chemopreventive agent in patients with high-risk or premalignant lesions reported that, among patients with oral leukoplakia, one of seven developed malignancy, while two showed histologic improvement. The study also demonstrated that oral curcumin, administered at doses up to 8000 mg/day for three months, was well tolerated and did not produce significant toxicity [281].

A phase II trial in patients with oral leukoplakia showed that curcumin induced a clinical response in 67.5% of patients compared with 55.3% in the control group, with 16 patients achieving a complete response. However, even though curcumin was well tolerated and demonstrated a significantly better clinical outcome, it did not improve histologic response, and continued treatment provided no additional benefit in patients with partial responses [282].

Compared with other treatment modalities, curcumin has demonstrated inconsistent outcomes in the management of OSMF and OLP. For instance, compared with lycopene or triamcinolone acetonide, studies have reported both comparable efficacy and inferior results. These discrepancies may be attributable to variations in dosage, formulation, and routes of administration across studies. Furthermore, the combined use of systemic and topical curcumin may offer greater therapeutic benefits than either modality alone. Therefore, future comparative studies should investigate different curcumin formulations and administration strategies, including combination approaches, and evaluate their efficacy against current standards of care for OSMF and OLP treatment. Clinical trials in patients with oral leukoplakia have demonstrated good, although limited, results, which suggests that different modalities or combinations containing curcumin should be explored. Nonetheless, curcumin demonstrates significant therapeutic potential for the management and chemoprevention of OPMDs such as oral leukoplakia, OLP, OSMF, erythroplakia, and oral graft-versus-host disease, with benefits in symptom relief and functional outcomes.

## 6. Pharmacokinetic Limitations and Strategies to Enhance Bioavailability of Curcumin

The oral bioavailability of curcumin is extremely low due to a number of factors, such as poor water solubility, rapid metabolism and intestinal excretion. Less than 1% of the ingested dose reaches systemic circulation. In order to improve curcumin’s problems, strategies have been developed, such as complexation with phospholipids and cyclodextrins, formulations with piperine, which inhibits hepatic glucuronidation, and the development of nanopreparations, such as liposomes, polymeric nanoparticles, dendrimers, inorganic nanoparticles, nanoemulsions and nanosuspensions. These approaches improve absorption, increase plasma half-life and potentiate therapeutic effects [19,283]. For instance, liposomes protect the compound and allow controlled release, while polymeric nanoparticles, such as PLGA and chitosan, provide sustained release and the possibility of active targeting [284].

In the context of oral cancer, the pharmacokinetic optimization of curcumin is essential in order to achieve effective concentrations in tumor tissues and obtain consistent clinical results (Table 2).

Thus, several mucoadhesive emulgel systems containing curcumin were tested, and the emulgel formed by poloxamer 407, Carbopol 974P^®^ and sesame oil showed better incorporation of curcumin in the nanoparticle core, preventing the formation of curcumin precipitates while also presenting higher stability. In addition, Carbopol 974P^®^ and sesame oil present higher viscosity, which allows for stronger structuration. This formulation was then selected for further investigation. However, it showed a poor cumulative retention profile. In the Cal27 and FaDu cell lines, as well as in normal cells, 70% of the incorporated curcumin was released within 24 h. The formulation reduced the IC_50_ in Cal27 cells, while the IC_50_ increased in both FaDu and normal cells. As expected, the efficacy of the formulation decreased when tested in an oral mucosa model; however, it still reduced Cal27 cell viability more effectively than that of normal cells [299]. A similar system comprising poloxamer 407 and Carbopol 974P^®^ containing curcumin achieved complete drug release after 8 h. Compared with free curcumin, the formulation resulted in higher IC_50_ values in FaDu cells and the normal oral cell line FNB6, but not in Cal27 cells. Consequently, this formulation may have limited efficacy as a primary therapeutic agent due to its slow drug release, ex vivo retention of curcumin in the mucosa, and the observed cytotoxicity profile [303].

Curcumin loaded in a lipid nanoemulsion of 100 nm presented higher bioavailability than nanoemulsions of 50 or 200 nm. The nanoemulsion led to cell proliferation inhibition by increasing miR-199a expression, which in turn targets PI3K, reducing its expression and the activation of its downstream targets such as Akt and mTOR [300].

A microgranular curcumin formulation led to higher curcumin serum levels than encapsulated curcumin in HNSCC patients. It showed significant reduction of FGF-2, which is involved in angiogenesis, after 4 h of treatment; reduction of GM-CSF between 15 min and 2 h; and reduction of IL-17 at the 1 h mark [301].

Injectable polydopamine/curcumin dual-modified polylactic acid/polycaprolactone coaxial staple fibers considerably increased ROS production, caspase-3 expression and consequently, cell death in vitro. This formulation, combined with laser irradiation in vivo, reduced tumor growth more effectively than the other formulations tested. Moreover, through bioinformatical analysis, a protein–protein interaction network was constructed, and five curcumin core targets were identified: STAT3, EGFR, BCL-2, AKT1, and GSK-3β, suggesting that curcumin exerts its anticancer effect in OSCC by targeting these proteins [298].

The curcumin analog AC17 exhibited higher cytotoxicity than curcumin in the Cal27 oral cancer cell line. AC17 induced a greater increase in apoptosis and a stronger reduction in cell migration compared to curcumin, while also increasing the proportion of cells in the S and G2 phases, suggesting cell-cycle arrest. Mechanistically, AC17 was shown to activate and upregulate FOXO3, a transcription factor involved in the regulation of cell growth, survival, and oxidative stress responses. FOXO3 activation subsequently increased the expression of its downstream targets p21 and GADD45A, leading to the downregulation of CDK1 and CDK2 and thereby inhibiting cell proliferation. However, AC17 reduced the viability of normal cells more effectively than Cal27 cells, indicating limited tumor selectivity. In this sense, soluble hyaluronic acid microneedles containing AC17 were assessed to promote AC17 tumor targeting. DMSO was also used to increase AC17 solubility. The AC17 microneedles showed a rapid dissolution rate, efficient drug delivery and good biosafety, and in vivo, led to a more pronounced reduction in tumor growth compared to free AC17. Consistent with in vitro findings, microneedle-mediated delivery led to increased expression of FOXO3, p21, and GADD45A, along with decreased CDK1 and CDK2 expression, whereas free AC17 only significantly upregulated p21 and downregulated CDK1 [85].

Nanocurcumin, consisting of curcumin incorporated in polyvinylpyrrolidone-stabilized nanoparticles, exhibited enhanced bioavailability and showed greater selectivity toward oral cancer cells compared with acetone-dissolved curcumin. It significantly reduced the percentage of viable cells by promoting apoptosis while simultaneously exerting antioxidant effects. Additionally, nanocurcumin inhibited cell migration more effectively than acetone-dissolved curcumin [297].

Curcumin-loaded chitosan-coated polycaprolactone nanoparticles were evaluated using chitosan of different molar masses, and low-molar-mass chitosan exhibited superior permeability compared with medium- and high-molar-mass formulations, even though it was not statistically significant. Although curcumin-loaded nanoparticles reduced cell viability, their cytotoxic effect was less pronounced than that of free curcumin. Notably, after 24 h of treatment, the nanoparticles containing curcumin led to increased viability, whereas a reduction in viability was only observed after 48 h of exposure [296].

A similar drug release profile was observed for curcumin-loaded lipid-core nanocapsules and chitosan-coated curcumin-loaded lipid-core nanocapsules. Moreover, both nanocapsules showed similar cytotoxicity but were less effective than curcumin alone. Interestingly, chitosan-coated blank lipid-core nanocapsules led to higher cell viability reduction after 48 h and 144 h than the nanocapsules containing curcumin. In addition, both nanosystems were incorporated in a hydrogel formulation that increased mucoadhesion and curcumin permeation; however, the hydrogel’s cytotoxicity was not evaluated [295].

Furthermore, curcumin-loaded PLGA nanoparticles decreased cell viability of a Cal27-cisplatin-resistant cell line but not of normal HGF nor of normal human oral keratinocyte cells. The nanoparticles containing curcumin increased ROS production; downregulated multiple drug resistance protein 1 and BCL-2; upregulated BAX, cytochrome c, Apaf-1, Endo G and AIF; and activated caspase-3 and -9, resulting in apoptosis induction [294].

Bovine serum albumin-coated gadolinium oxide nanoparticles loaded with curcumin exhibited high drug-loading capacity and good biocompatibility while demonstrating slightly higher cytotoxicity than free curcumin against RPMI 2650 cells [293].

In a HNSCC cell line, curcumin-loaded mesoporous silica nanoparticles led to a slight decrease in IC_50_ compared to free curcumin. The nanoparticles reduced BCL-2 expression while increasing BAX and ROS production [290]. In Sprague Dawley rats, mesoporous nanoparticulates containing curcumin showed increased drug permeability and drug release, resulting in increased bioavailability of curcumin. This formulation was designed for oral cancer treatment and might be a promising therapeutic strategy for this disease [291].

Curcumin-loaded bacterial cellulose/alginate/gelatin composite films demonstrated selective cytotoxicity toward the OSCC line Cal27 compared with normal cells, leading to a significant reduction in cancer cell viability. In addition to their anticancer selectivity, these composite films also exhibited antibacterial activity against *E. coli* and *S. aureus* [292].

Several liposomal formulations containing curcumin, such as multilamellar vesicles, small unilamellar vesicles and ethanol injection vesicles, were tested in the SCC-9 cell line. Multilamellar vesicles exhibited the highest encapsulation efficiency, whereas ethanol injection vesicles presented the most favorable release profile and the lowest IC_50_. These results suggest that vesicle size influenced cytotoxicity, as the larger multilamellar vesicles displayed reduced anticancer activity compared with smaller formulations. It is possible that smaller vesicles facilitate faster drug release and enhance cellular uptake. Nonetheless, other factors, including the encapsulated drug, the number of lamellae, and the composition of the delivery system, may also play an important role in drug release [289].

Small extracellular vesicles isolated from Jurkat cells containing curcumin showed good loading efficiency and led to migration and metabolic activity inhibition slightly more effectively than free curcumin in vitro. In a 4-NQO mouse model, it also reduced tumor burden more effectively than free curcumin. Nonetheless, biodistribution and pharmacokinetic analysis, as well as the immunological effects of these vesicles, need to be elucidated [305].

Liposomal nanoparticles encapsulating curcumin showed good encapsulation efficiency, relative stability, and released approximately 58% of the drug within 48 h. However, contrary to the authors’ interpretation, encapsulated curcumin exhibited a higher IC_50_ compared to free curcumin, indicating reduced cytotoxicity [288].

Nanoparticles coated with tumor-targeting biotin containing disulfide-linked polycurcumin and the photothermal agent T780 were assessed in HNSCC cells and led to reduction of cell viability, clonogenic ability and increased apoptosis more effectively than free curcumin, polycurcumin or T780. Treatment with the nanoparticle’s formulation increased oxidative stress and lipid peroxidation levels, suggesting that its cytotoxic effects are correlated with ROS accumulation and the induction of ferroptosis. The nanoparticles also appeared to suppress tumor cell stemness, as evidenced by reduced expression of stemness-associated markers, including zinc finger E-box–binding homeobox 1, Nanog, c-Myc, Notch, and Patched. In addition, nanoparticle treatment promoted immunogenic cell death, as indicated by increased calreticulin (CRT) externalization and high-mobility group box 1 (HMGB1) release, suggesting enhanced tumor immunosensitization. Nanoparticle administration markedly reduced tumor volume and weight, decreased tumor recurrence, suppressed tumor stemness, and significantly prolonged survival in vivo. Consistent with the in vitro findings, tumors exhibited increased CRT exposure and HMGB1 release, enhanced infiltration of CD4^+^ and CD8^+^ T cells, a significant reduction in Treg cells, increased percentage of mature dendritic cells, and polarization of macrophages from the M2 to the pro-inflammatory M1 phenotype [302].

Curcumin-loaded niosome injections and niosome-based curcumin mouthwash were evaluated in a 4-NQO mouse model, showing that only the injections significantly prevented the development of severe forms of dysplasia [287].

Curcumin-loaded liposomes were shown to reduce cell viability less effectively than curcumin dissolved in DMSO in OSCC cell lines. The liposomes led to reduced NF-κB activation but not through interference with Akt activity. Moreover, the liposomes containing curcumin decreased COX-2, MMP-9, cyclin D1, BCL-2, BCL-xL, MCL-1L and MCL-1S expression. The curcumin-loaded liposomes led to a significant reduction in tumor growth and NF-κB expression in vivo [304].

Moreover, both liposomes loaded with curcumin and liposome-encapsulated CDF combined with cisplatin led to increased cell death in UM-SCC-1 cells while also increasing cell death of CD44^hi^, a marker of cell stemness, cells. Cisplatin-resistant UM-SCC-1 cells showed higher sensitivity to the CDF formulation, which also decreased cells expressing CD44. In addition, CDF liposomes, in combination with cisplatin, reduced tumor growth and CD44 expression in in vivo models with cisplatin-resistant UM-SCC-1 [54].

In the SCC-4 cell line, κc/XG mucoadhesive nanocomposite sponges loaded with tetrahydrocurcumin nanocrystals or tetrahydrocurcumin were evaluated, and the sponges loaded with tetrahydrocurcumin nanocrystals showed better drug release. Tetrahydrocurcumin nanocrystals alone led to enhanced cytotoxicity compared to free tetrahydrocurcumin. The nanocomposites with tetrahydrocurcumin nanocrystals, in vivo, led to oral precancerous lesions receding to simple leukoplakia, while some oral precancerous lesions progressed when treated with nanocomposites loaded with tretrahydrocurcumin. Furthermore, nanocomposites containing tetrahydrocurcumin nanocrystals repressed cyclin D1 expression [286].

Tetrahydrocurcumin-phytosomes exhibited improved aqueous solubility, with complete drug release observed within 1 h. In vitro, the phytosomal formulation showed a higher IC_50_ than tetrahydrocurcumin in solution; however, it demonstrated greater selectivity toward oral cancer cells compared with normal cells after 48 h of treatment. Moreover, tetrahydrocurcumin-phytosomes inhibited cell migration and colony formation more effectively than tetrahydrocurcumin in solution and cisplatin. After 48 h of treatment, apoptosis induction was enhanced relative to free tetrahydrocurcumin, accompanied by increased BAX and caspase-8 expression and a reduction in oxidative stress [285].

Besides these formulations, several others exist that can improve curcumin stability and bioavailability and have been explored in other cancer types. For instance, cyclodextrin-based nanogels improve curcumin’s stability in aqueous solutions, while a Nα-9-fluorenylmethoxycarbonyl-diphenylalanine (Fmoc-FF) peptide-based nanogel containing curcumin showcased good stability and a sustained release profile [306,307]. However, the Fmoc-FF nanogel reduced the nuclear internalization rate of curcumin and curcumin’s cytotoxicity in thyroid cancer cells [307]. On the contrary, a different nanogel also containing curcumin was efficiently internalized by both prostate and breast cancer cells, leading to increased cytotoxicity when compared to free curcumin [308]. Nanogels are easier to produce and can enhance drug accumulation at target sites and substantially reduce the exposure of curcumin to serum proteins and biological degradation following systemic administration and could be an interesting platform to test in HNSCC [308].

In summary, some of the studies analyzing nanoformulations, emulsion gels and other types of formulations did not compare their effects with the free compounds, which does not allow for a clear assessment of their potential improvements. Nonetheless, the results of the studies that explored these types of formulations seem to point to variable outcomes. For instance, some formulations improved cytotoxicity in some cell lines while reducing it in others. Moreover, the size of the particle affects permeation, drug release and encapsulation efficiency. Thus, the tumor characteristics, the type of formulation and the compound used need to be taken into account to improve the antitumoral effects of curcumin and its analogs and to attenuate the issue of curcumin’s bioavailability that limits its clinical use. Despite the growing interest in nanoformulations and the substantial body of preclinical research generated in recent years, no nanoformulation has yet received approval for the treatment of HNSCC. This underscores the difficulties in predicting the clinical performance of these technologies and successfully translating promising experimental findings into therapeutic applications. Notably, no clinical trials evaluating curcumin-based nanoformulations have been conducted in patients with HNSCC, despite encouraging preclinical evidence supporting their therapeutic potential. In contrast, a clinical study in patients with localized muscle-invasive bladder cancer reported improved complete clinical response rates following treatment with nanocurcumin in combination with chemotherapy. Although these improvements did not reach statistical significance, this may have been attributable to the limited sample size of the study [309]. Nonetheless, these findings highlight the need for clinical investigations assessing curcumin-based nanoformulations in patients with HNSCC to determine whether the promising preclinical results can be translated into meaningful clinical benefits.

## 7. Role of Curcumin in Patients’ Quality of Life

Curcumin and its analogs have also been assessed to try to improve patients’ quality of life by attenuating secondary effects of cancer treatment. For instance, in Detroit 562 cells, short-term exposure (4 h) to low concentrations of curcumin produced minimal or no bactericidal effects, whereas treatment with 200 μM of curcumin resulted in near-complete eradication of the tested microbial species. Moreover, a 30 min exposure to curcumin significantly decreased the expression of the pro-inflammatory mediators IL-8, IL-6, VEGF, GM-CSF, monocyte chemoattractant protein-1 (MCP-1), and TNF-*α*, an effect that could be reproduced upon repeated treatment. These effects suggest that curcumin might have therapeutic potential for the treatment or prevention of oral mucositis [310]. Natural and synthetic curcumin were compared in an in vitro model of oral mucositis, and no significant differences were observed in their cytotoxic or bactericidal effects or in their impact on bacterial adherence and invasion, while both also reduced the secretion of IL-6, IL-8, GM-CSF, VEGF, MCP-1, and TNF-α to similar levels. Nonetheless, synthetic curcumin offers practical advantages, as it is odorless and tasteless and shows improved solubility in DMSO [311].

Curcumin fortified with piperine was investigated in HNC patients undergoing radiochemotherapy to prevent mucositis. The study found that the formulation delayed onset and reduced the incidence and grade of mucositis while increasing the percentage of patients who completed radiotherapy [312].

Nanomicelle curcumin capsules were also evaluated for the prevention of radiotherapy-induced oral mucositis in HNC patients. Treatment with nanomicelle curcumin delayed the onset and reduced the severity of mucositis, mitigated weight loss, and prevented grade 4 mucositis, which occurred in 50% of patients in the control group [313].

A curcumin-based oral gel was assessed as a potential preventive treatment for oral mucositis in primary oral keratinocytes, immortalized oral keratinocytes, dysplastic oral keratinocytes and OSCC cells. OSCC cells were less sensitive to the curcumin gel, while immortalized oral keratinocytes were the most sensitive to hydrogen peroxide. Pretreatment of all cell lines with non-cytotoxic concentrations of the curcumin gel prior to hydrogen peroxide exposure significantly reduced ROS production compared with untreated controls, showcasing the potential of the curcumin gel in preventing oral mucositis [314]. In HNC patients undergoing concurrent chemoradiation, a curcumin oral gel significantly reduced TNF-α and IL-6 salivary levels and the grade of mucositis compared to the control [315]. Moreover, HNC patients treated with curcumin gel show increased EGF salivary levels after radiation treatment, which correlated with prevention and reduction of oral mucositis severity and associated pain [316]. A similar study found that six weeks after treatment with curcumin gel, patients exhibited increased salivary EGF levels; a decrease, although not statistically significant, in salivary LDH levels; and a reduction in both the grade and severity of oral mucositis compared with controls [317].

Curcumin mouth rinse was compared with chlorhexidine, which is an antimicrobial biguanide, mouthwash in HNC patients for the management of radiochemotherapy-induced oral mucositis. The results showed that curcumin mouth rinse reduced erythema, ulceration, oral mucositis severity, and associated pain more effectively than chlorhexidine mouthwash [318]. A similar study exploring curcumin mouthwash showed that the risk of radiation-induced oral mucositis onset was 50% lower and also significantly delayed compared with a benzydamine mouthwash [319]. Curcumin mouthwash and curcumin nanocapsules have also been studied for amelioration of radiotherapy-induced oral mucositis, and both treatment options showed similar results that led to reduction of pain and burning sensation compared to the placebo group. Nonetheless, 33% of patients receiving curcumin mouthwash remained ulcer-free versus only 15% in the group receiving curcumin nanocapsules [320].

Turmeric extract was also found to reduce the incidence and severity of radiotherapy-induced oral mucositis in HNC patients, while a turmeric mouthwash delayed and decreased radiation-induced oral mucositis and reduced intolerable mucositis and changes in body weight compared to povidone-iodine mouthwash [321,322]. In another study comparing turmeric mouthwash with benzydamine mouthwash, although symptom severity progressively worsened from week 1 to week 7 in both groups, patients treated with turmeric mouthwash consistently demonstrated better outcomes than those receiving benzydamine in terms of the severity of treatment-induced oral mucositis and associated symptoms, including oral pain, speech difficulties, eating and drinking limitations, dysphagia, and taste alterations [323]. Moreover, turmeric mouthwash was also reported to have better efficacy than saline mouthwash on treatment-induced oral mucositis [324].

Bio-enhanced turmeric formulation capsules tested in oral cancer patients with radiation-induced oral mucositis showed reduction of pain, oral mucositis, dysphagia and dermatitis severity while reducing body weight loss compared with the control [325].

A *Curcuma longa* gel was also investigated for its protective effects on radiation-induced oral mucositis in patients with HNC, and it improved mucositis grade and decreased size of oral lesions [326].

The protective role of nanocurcumin in reducing acute radiation toxicity in locally advanced HNC patients was assessed, and no significant improvement was observed regarding mucous membrane, skin and salivary gland toxicity and thrombocytopenia. Nonetheless, nanocurcumin improved pharyngeal/esophageal and laryngeal toxicity and reduced neutropenia, leukopenia and anemia severity [327].

Furthermore, curcumin solubilized in DMSO was investigated for its potential to mitigate radiotherapy-induced side effects in rats subjected to laryngeal irradiation. This treatment was associated with significant downregulation of TNF-α, as well as significant reduction in edema, hyperemia, necrosis, and pseudostratification compared with radiotherapy alone [328].

Cancer cachexia is a progressive condition that encompasses three clinically relevant stages—precachexia, cachexia, and refractory cachexia—although not all patients progress through every stage. Precachexia is characterized by early metabolic and clinical alterations, such as anorexia and impaired glucose tolerance, occurring before significant weight loss (≤5%). Cachexia is defined by a weight loss exceeding 5% over six months, or ongoing weight loss greater than 2% in individuals with a BMI below 20 kg/m^2^ or with sarcopenia, provided the refractory stage has not been reached. Refractory cachexia occurs in the context of advanced, treatment-resistant disease and is marked by active catabolism, poor functional status, and a life expectancy of less than three months. At this stage, nutritional interventions offer limited benefit, and care focuses on symptom management and improving quality of life [329,330].

In this sense, effective management of cancer cachexia is critical, as it is associated with increased mortality. Thus, in patients with locally advanced or metastatic HNSCC, curcumin was evaluated for the treatment of cancer anorexia–cachexia, and it significantly improved muscle mass and showed a trend toward preservation of body fat mass and basal metabolic rate. Additionally, although not statistically significant, curcumin attenuated the decline in handgrip strength and absolute lymphocyte count, while exhibiting a toxicity profile comparable to placebo [329]. Further analysis of the same study showed that curcumin significantly improved physical and emotional functioning, reduced pain, and decreased appetite loss. Nonetheless, no significant changes were observed in global health status or in role, cognitive and social functioning. In addition, curcumin did not improve fatigue, nausea and vomiting, dyspnea, insomnia, constipation, or diarrhea compared to the placebo group [331].

Moreover, curcumin at a high dose increased apoptosis and reduced colony formation capabilities of HN4 cells compared with the control, while it improved physical strength, food intake, weight, abdominal bloating and insomnia compared to the placebo group. The curcumin group showed the smallest number of patients in all the major adverse events observed. Curcumin was also found to inhibit S100A9, a protein involved in the regulation of inflammatory processes and immune response, to a certain extent, while when combined with a S100A9 inhibitor, it led to enhanced DR5, DR4, cyclin B1 and p21 expression; cleavage of PARP and caspase-9; apoptosis induction; and reduction of the lipid mobilization factors, hormone-sensitive lipase and adipose triglyceride lipase. Thus, it is suggested that curcumin might be effective for the treatment of cancer anorexia–cachexia [332].

In conclusion, these results show that curcumin is effective in ameliorating and preventing oral mucositis and its symptoms. Furthermore, curcumin mouthwash is more effective in the prevention and treatment of oral mucositis than other mouthwashes. Other formulations and compounds, such as turmeric, also seem to be beneficial for the treatment of this condition. Similar observations were made for the treatment of cancer anorexia–cachexia, for which every study showed improvement of symptoms. Thus, it seems that curcumin can improve quality of life in patients with HNC by preventing and attenuating oral mucositis, radiation toxicity and cancer anorexia–cachexia.

## 8. Conclusions

Curcumin and its derivatives demonstrate potential as anticancer compounds in HNSCC, increasing cell death and modulating key molecular pathways involved in tumor progression and proliferation. Despite promising preclinical evidence, clinical validation remains limited, highlighting the need for well-designed trials and optimized delivery strategies. A major challenge for clinical translation lies in the unfavorable pharmacokinetic profile of curcumin, characterized by low systemic availability, extensive biotransformation, and limited persistence in circulation, which collectively restrict its therapeutic applicability. In addition, careful consideration must be given to the relationship between experimental dosing and clinically realistic exposure levels, as many in vitro studies rely on concentrations that are unlikely to be reproduced in patients, particularly in systemic contexts.

From a clinical standpoint, curcumin-based approaches have so far been explored mainly in supportive care contexts, particularly in the management of treatment-associated toxicities and in improving patient-reported outcomes, while robust evidence supporting direct antitumor effects in humans remains limited. In this context, nano-based delivery systems may offer more than incremental physicochemical improvements, as they can enhance stability and systemic exposure and may enable biologically relevant concentrations that are otherwise difficult to achieve with free curcumin, thereby increasing their translational relevance.

Future research should focus on translating these findings into effective, safe, and targeted therapeutic regimens by exploring novel compounds, nanoformulations, and interactions with the tumor microenvironment, as well as combination approaches with conventional anticancer therapies and other therapeutic agents, which are already emerging as promising strategies to enhance treatment efficacy and overcome resistance mechanisms. In addition, given the distinct molecular and clinical behavior of HPV-positive and EBV-associated head and neck cancers, future studies should consider these viral subtypes as biologically relevant stratification factors when evaluating curcumin-based interventions. Although direct evidence remains limited, the overlap between virus-driven oncogenic pathways and the molecular targets of curcumin suggests potential subtype-specific effects that warrant further investigation.

Nonetheless, curcumin seems to show a higher potential as a therapeutic option to attenuate adverse events associated with radiotherapy and to improve the quality of life of HNSCC patients.

## Figures and Tables

**Table 2:** Curcumin and analog-based nanoformulations in HNSCC: therapeutic potential and drawbacks.

Formulation	Delivery Platform	Main Advantages	Biological Outcome	Main Disadvantages	References
Liposomal curcumin and liposomal CDF	Liposome	Higher cytotoxicity than liposomal curcumin	CDF liposomes were more cytotoxic than liposomal curcumin in cisplatin-resistant OSCC cells and led to reduced growth in vivo when combined with cisplatin. Nonetheless, both formulations resensitized cells to cisplatin.	-	[54]
AC17-loaded dissolvable microneedles	Hyaluronic acid microneedle patch	Rapid drug release and good biocompatibility and biosafety; higher cytotoxicity than free AC17	AC17 showed better efficacy than curcumin. Moreover, AC17 microneedles led to higher tumor growth inhibition in vivo compared to free AC17.	The microneedles only allow for localized delivery	[85]
Tetrahydrocurcumin-phytosomes	Phytosomes	Improved aqueous solubility, cellular uptake and cumulative drug released, with complete drug release observed within 1 h, and greater selectivity toward oral cancer cells after 48 h of treatment	The phytosomes showed higher IC_50_ than free tetrahydrocurcumin both in oral cancer and normal cell lines. Nonetheless, the phytosomes were more effective in inhibiting migration and colony formation and in promoting apoptosis.	Lower cytotoxicity	[285]
Tetrahydrocurcumin-loaded sponges and tetrahydrocurcumin nanocrystal-loaded sponges	Mucoadhesive nanocomposite κ-carrageenan/xanthan gum sponges	Tetrahydrocurcumin nanocrystal sponges showed improved solubility and a good drug release profile	Tetrahydrocurcumin nanocrystals led to increased cell viability reduction compared to free tetrahydrocurcumin. In vivo, tetrahydrocurcumin nanocrystal-loaded sponges improved oral lesions more effectively than tetrahydrocurcumin-loaded sponges.	-	[286]
Curcumin-loaded niosome in the form of mouthwash or injection	Niosome nanocapsule	Great encapsulation efficiency (98%)	Curcumin niosome injections prevented the development of severe forms of dysplasia, while the mouthwash showed no significant difference compared to the negative control	-	[287]
Curcumin encapsulated liposomal nanoparticles	Liposomal nanoparticles	Good encapsulation efficiency (82%), sustained release profile, increased aqueous solubility and stability	The liposomal nanoparticles containing curcumin led to higher IC_50_ in OSCC cells compared to free curcumin	Lower cytotoxicity	[288]
Several curcumin-loaded liposome formulations	Multilamellar vesicles, small unilamellar vesicles and ethanol injection vesicles	Smaller liposomal systems have increased cellular uptake and drug release, and potentially enhanced bioavailability and cytotoxicity. Bigger systems have higher encapsulation efficiency.	Multilamellar vesicles exhibited the highest encapsulation efficiency, while ethanol injection vesicles the most favorable release profile and the lowest IC_50_	Bigger liposomal systems have lower cytotoxicity; smaller systems have lower encapsulation efficiency	[289]
Curcumin-loaded mesoporous silica nanoparticles	Mesoporous silica nanoparticles	Drug-loaded efficiency of 82–83% with sustained curcumin release over 24 h. Slight improvement of cytotoxicity in vitro. Increased solubility, drug permeability, drug release, and bioavailability in vivo.	The nanoparticles led to a slightly decreased IC_50_ compared to free curcumin and increased apoptosis induction in vitro	-	[290,291]
Curcumin-loaded bacterial cellulose/alginate/gelatin composite films	Multifunctional biopolymer composites	Hydrated curcumin-loaded films showed improved tensile strength and Young’s modulus in comparison to nanocurcumin-loaded ones	Curcumin-containing composite films showed selective cytotoxicity toward Cal27 cells compared with normal cells	Incorporation of curcumin led to lower rate of water absorption, decreased adhesive performance, elongation at break and mechanical properties	[292]
Bovine serum albumin-coated gadolinium oxide nanoparticles containing curcumin	Gadolinium oxide nanoparticles	Drug release occurred in a sustained and pH-dependent manner. The nanoparticles exhibited excellent biosafety and biocompatibility.	Slightly higher cytotoxicity than free curcumin against RPMI 2650 cells, although not statistically significant	-	[293]
Curcumin nanoparticles	PLGA nanoparticles	Greater water solubility and systemic bioavailability	The curcumin nanoparticles led to reduced cell viability of oral cancer cells but not of normal cells	-	[294]
Curcumin-loaded lipid-core nanocapsules coated or uncoated with chitosan incorporated or not in a hydrogel	Lipid-core nanocapsules	Chitosan coating does not interfere with the drug release profile. The hydrogel increased mucoadhesion while increasing curcumin permeation.	Curcumin-loaded nanocapsules and chitosan-coated curcumin-loaded nanocapsules showed similar cytotoxicity but were less effective than curcumin alone. The nanocapsules incorporated in the hydrogel were not evaluated for their cytotoxicity.	Chitosan coating did not improve mucoadhesive potential of nanosystems; lower cytotoxicity	[295]
Curcumin-loaded chitosan-coated nanoparticles	Chitosan-coated polycaprolactone nanoparticles	Encapsulation efficiency of >99% and good mucoadhesive properties	Curcumin-loaded nanoparticles reduced cell viability only after 48 h of exposure and showed less efficacy than free curcumin	Lower cytotoxicity	[296]
Nanocurcumin	Polyvinylpyrrolidone-stabilized nanoparticles	Good biocompatibility and improves stability and bioavailability; higher cytotoxicity	Nanocurcumin showed greater selectivity toward oral cancer cells and lower IC_50_, and inhibited migration more effectively than acetone-dissolved curcumin	-	[297]
Injectable polydopamine/curcumin dual-modified polylactic acid/ polycaprolactone coaxial staple fibers	Staple fibers	Good biocompatibility. Polydopamine allows for photothermal effects.	The injection of the polydopamine/curcumin dual-modified polylactic acid/ polycaprolactone coaxial staple fibers in combination with laser irradiation led to improved cytotoxicity when compared with polydopamine or curcumin polylactic acid/ polycaprolactone coaxial staple fibers	The addition of polydopamine reduced drug release	[298]
Poloxamer 407, acrylic acid derivatives and oil phase emulgel systems containing curcumin	Mucoadhesive emulgel systems	The emulgel formed by poloxamer 407, carbopol 974P and sesame oil showed higher stability and better incorporation of curcumin in the nanoparticle core, preventing the formation of curcumin precipitates. Carbopol 974P and sesame oil present higher viscosity, which allows for stronger structuration.	The formulation reduced the IC_50_ in Cal27 cells, while the IC_50_ increased in both FaDu and normal cells compared to free curcumin. The effects were worse in an oral mucosa model, but the emulgel still reduced Cal27 viability more effectively than that of normal cells	Poor cumulative retention profile; variable cytotoxic effects	[299]
Curcumin nanoemulsion	Lipid nanoemulsion	Greater bioavailability	The curcumin nanoemulsion reduced cell proliferation by increasing miR-199a expression, inhibiting the PI3K pathway signaling	-	[300]
Transmucosal administration of a curcumin microgranular formulation	Microgranular formulation	Transmucosal administration of curcumin promotes prolonged contact with oral mucosa and direct absorption	Higher curcumin serum levels than encapsulated curcumin in HNSCC patients	-	[301]
Biotin nanoparticles containing disulfide-linked polycurcumin and T780	DSPE–PEG–biotin-coated nanoparticles	The coating with DSPE–PEG–biotin enables tumor targeting. Moreover, the nanoparticle takes advantage of the high intratumoral glutathione levels, leading to the disassembly of the nanoparticle and the release of both drugs; higher cytotoxicity.	The nanoparticles reduced cell viability and clonogenic ability and increased apoptosis more effectively than free curcumin, polycurcumin or T780. Moreover, they reduced tumor volume and significantly prolonged survival in vivo.	-	[302]
Poloxamer 407 micelles with Carbopol 974P^®^ incorporated containing curcumin	Mucoadhesive micelles	The formulation displays viscoelastic properties, good retention and increased mucoadhesive force	The formulation resulted in higher IC_50_ values in FaDu cells and normal cells, but not in Cal27 cells, compared with free curcumin	Could not permeate porcine oral mucosa and showed a slow release profile, even though the release was complete after 8 h; variable cytotoxicity	[303]
Liposomal curcumin	Liposome	-	Curcumin-loaded liposomes reduced cell viability less effectively than curcumin dissolved in DMSO. Nonetheless, in vivo curcumin liposomes significantly reduced tumor growth.	Lower cytotoxicity	[304]
Jurkat cells small extracellular vesicles containing curcumin	Small extracellular vesicles	Good loading efficiency; higher cytotoxicity	The vesicles inhibited migration and metabolic activity slightly more effectively than free curcumin in vitro. They also reduced tumor burden more effectively than free curcumin in vivo.	-	[305]

## Data Availability

No new data were created or analyzed in this study. Data sharing is not applicable to this article.

## Abbreviations

The following abbreviations are used in this manuscript:

AIFP	Apoptosis-inducing factor
AMPK	AMP-activated protein kinase
BAD	BCL-2-associated death promoter
BCL-2	B-cell lymphoma 2
BCL-xL	B-cell lymphoma-extra large
BCRP	Breast cancer resistance protein
BDMC	Bis-demethoxycurcumin
CDC	Cell division cycle
CDK	Cyclin-dependent kinase
CDF	Curcumin-difluorinated
cIAP	Cellular inhibitor of apoptosis
COX	Cyclooxygenase
COL1A1	Collagen type I alpha 1 chain
CRT	Calreticulin
CTGF	Connective tissue growth factor
DBA	Dibenzylideneacetones
DMC	Demethoxycurcumin
E2R4	E2 receptor 4
EGFR	Epidermal growth factor receptor
EMT	Epithelial–mesenchymal transition
ERK	Extracellular signal-regulated kinase
FAK	Focal adhesion kinase
FOXO	Forkhead box O
FOSL1	FOS-like antigen 1
GM-CSF	Granulocyte–macrophage colony-stimulating factor
HMGB1	High-mobility group box 1
HNC	Head and neck cancer
HNSCC	Head and neck squamous cell carcinoma
HPV	Human papillomavirus
IGFBP-5	Insulin-like growth factor binding protein-5
IL	Interleukin
iNOS	Nitric oxide synthase
JNK	c-Jun N-terminal kinase
LPS	Lipopolysaccharide
LTBP2	Latent-transforming growth factor β-binding protein 2
MAPK	Mitogen-activated protein kinase
MCP-1	Monocyte chemoattractant protein-1
MMP	Matrix metalloproteinase
MOMI	Modified oral mucositis index
NFC	Nasopharyngeal carcinoma
NF-κB	Nuclear factor κB
NO	Nitric oxide
Nrf2	Nuclear factor erythroid 2-related factor 2
NSCLC	Non-small cell lung cancer
OLP	Oral lichen planus
OPMDs	Oral potentially malignant disorders
OSCC	Oral squamous cell carcinoma
OSMF	Oral submucous fibrosis
PARP	Poly-(ADP ribose) polymerase
PD-1	Programmed cell death 1
PD-L	Programmed cell death ligand
PI3K	Phosphatidylinositol 3-kinase
PKC	Protein kinase C
Rb	Retinoblastoma
ROS	ROS
SOD	Superoxide dismutase
SP1	Specific protein 1
STAT3	Signal transducer and activator of transcription 3
STR	Short tandem repeat
TGFβRII	Transforming growth factor-β receptor II
THC	Tetrahydrocurcumin
TLR	Toll-like receptor
TME	Tumor microenvironment
TNF	Tumor necrosis factor
TSCC	Tongue squamous cell carcinoma
VEGF	Vascular endothelial growth factor
XIAP	X-linked inhibitor of apoptosis protein
